# RDUR, a lncRNA, Promotes Innate Antiviral Responses and Provides Feedback Control of NF-κB Activation

**DOI:** 10.3389/fimmu.2021.672165

**Published:** 2021-05-14

**Authors:** Yuhai Chen, Jiayue Hu, Shasha Liu, Biao Chen, Meng Xiao, Yingying Li, Yuan Liao, Kul Raj Rai, Zhonghui Zhao, Jing Ouyang, Qidong Pan, Lianfeng Zhang, Shile Huang, Ji-Long Chen

**Affiliations:** ^1^ CAS Key Laboratory of Pathogenic Microbiology and Immunology, Institute of Microbiology, Chinese Academy of Sciences (CAS), Beijing, China; ^2^ Key Laboratory of Fujian-Taiwan Animal Pathogen Biology, College of Animal Sciences, Fujian Agriculture and Forestry University, Fuzhou, China; ^3^ College of Life Sciences, University of the Chinese Academy of Sciences, Beijing, China; ^4^ Institute of Laboratory Animal Science, Chinese Academy of Medical Sciences & Comparative Medical Center, Beijing, China; ^5^ Department of Biochemistry and Molecular Biology, Louisiana State University Health Sciences Center, Shreveport, LA, United States

**Keywords:** influenza A virus, long non-coding RNA, innate immunity, NF-κB, inflammation

## Abstract

Influenza A virus (IAV), a highly infectious respiratory pathogen, remains a major threat to global public health. Numerous long non-coding RNAs (lncRNAs) have been shown to be implicated in various cellular processes. Here, we identified a new lncRNA termed RIG-I-dependent IAV-upregulated noncoding RNA (RDUR), which was induced by infections with IAV and several other viruses. Both *in vitro* and *in vivo* studies revealed that robust expression of host RDUR induced by IAV was dependent on the RIG-I/NF-κB pathway. Overexpression of RDUR suppressed IAV replication and downregulation of RDUR promoted the virus replication. Deficiency of mouse RDUR increased virus production in lungs, body weight loss, acute organ damage and consequently reduced survival rates of mice, in response to IAV infection. RDUR impaired the viral replication by upregulating the expression of several vital antiviral molecules including interferons (IFNs) and interferon-stimulated genes (ISGs). Further study showed that RDUR interacted with ILF2 and ILF3 that were required for the efficient expression of some ISGs such as IFITM3 and MX1. On the other hand, we found that while NF-κB positively regulated the expression of RDUR, increased expression of RDUR, in turn, inactivated NF-κB through a negative feedback mechanism to suppress excessive inflammatory response to viral infection. Together, the results demonstrate that RDUR is an important lncRNA acting as a critical regulator of innate immunity against the viral infection.

## Introduction

Influenza A virus (IAV), a highly infectious respiratory pathogen that causes annual epidemics and occasional pandemics in humans and animals, has continued to be a top global public health threat. Seasonal influenza epidemics can result in 3-5 million severe cases and 300,000-500,000 deaths globally every year (1, 2). Host innate immune system provides immediate and the first line of defense against virus infection and the innate immunity is a formidable barrier to influenza virus (3). The pathogen associated molecular patterns (PAMPs) such as viral RNA present in infected cells are recognized as foreign substances by pattern recognition receptors (PRRs) (4–7). This intracellular detection of PAMPs by PRRs triggers signal cascades to induce the production of various cytokines and interferons (IFNs). IFNs in turn stimulate the expression of hundreds of genes known as IFN-stimulated genes (ISGs) and establish an antiviral state (8). Retinoic acid-inducible gene I (RIG-I)-like receptors (RLRs) and Toll-like receptors (TLRs) are major families of PRRs involved in sensing virus infection (9). RIG-I is crucial for recognition of viral RNA in the cytoplasm and for the production of type I and type III IFNs in the virus infected epithelial cells, alveolar macrophages and conventional dendritic cells (10). RIG-I interacts with mitochondrial antiviral signaling adaptor (MAVS) (11), leading to the activation of interferon regulatory factor 3 (IRF3) and nuclear factor-kappa B (NF-κB) (12, 13). As an important regulator of immunity and inflammation, NF-κB is mediated by multiple mechanisms and dysregulation of NF-κB pathway causes a wide range of disorders ranging from inflammation, autoimmune diseases to oncogenesis (14–16).

Although a variety of host factors have been shown to participate in IAV pathogenesis, the mechanisms underlying IAV-host interaction are still elusive. Long non-coding RNAs (lncRNAs), as a new group of host factors, are emerging as another critical layer modulating the host response to virus infection (17–19). Recently, increasing evidence has demonstrated the crucial roles of lncRNAs in many aspects of the innate and adaptive immunity (19–32). The broad-spectrum activities and versatile regulatory mechanisms of lncRNAs suggest that they may be key regulators in antiviral immunity (20, 21). Some lncRNAs such as lncRNA-Cox2, lncRNA-PACER, lncRNA-NEAT1 and lincRNA-EPS have been shown to regulate the inflammatory response through specific interactions with cellular proteins (24, 28). Importantly, several lncRNAs have been found to be involved in the pathogenesis of IAVs (30, 33, 34). For example, a lncRNA named negative regulator of antiviral response (NRAV) acts as a key regulator of host defense against viral infection, which negatively regulates the initial transcription of multiple ISGs during IAV infection (30). Silencing lncRNA#32 is able to reduce the expression levels of ISGs dramatically, resulting in increased sensitivity of host to encephalomyocarditis virus infection (35). On the other hand, some lncRNAs may favor viral infection and replication. For instance, a study has described that lncRNA-CMPK2, which is stimulated by IFNs through the JAK-STAT pathway, suppresses the expression of several antiviral ISGs (22). LncRNA-ACOD1, which can be induced by multiple viruses but not by IFNs, facilitates viral replication in mice (36). However, despite the increased number of functional lncRNAs involved in innate immunity, the importance of lncRNAs in IAV infection and pathogenesis remains largely unknown. Moreover, the specific functions of these lncRNAs and the precise mechanisms behind their actions in host antiviral immunity are poorly characterized.

In this study, we identified a new lncRNA termed RDUR, which was greatly induced by IAV and several other viruses. Both *in vitro* and *in vivo* data showed that RDUR acted as a key antiviral regulator in the host innate immunity, since disruption of mouse RDUR (mRDUR) expression profoundly promoted the virus replication and increased virulence in the animals infected with IAV. These observations suggest that upregulation of RDUR is critical for host immune response to IAV infection. Furthermore, we observed that robust expression of RDUR prevented the host from severe inflammation reaction *via* a mechanism possibly involving a negative feedback control of IAV-induced NF-κB activation and inflammation. Together, these results indicate that RDUR is an important host factor that functions as positive regulator of innate immunity against virus infection.

## Materials and Methods

### Ethics Statement

The animal experimental design and protocol used in this study were approved by the Research Ethics Committee of Institute of Microbiology, Chinese Academy of Sciences (Permit number SQIMCAS2015008). All mouse experimental procedures were performed in accordance with the Regulations for the Administration of Affairs Concerning Experimental Animals approved by the State Council of People’s Republic of China.

### Virus Strains

Influenza virus strains A/WSN/33 (H1N1), A/PR/8/34 (H1N1), CA04 (H1N1), A/Shanghai-Jiading/SWL1970/2015 (H1N1) (a seasonal H1N1 influenza A virus, provided by Prof. Dayan Wang from Chinese National Influenza Center, Beijing, China), Sendai virus (SeV) and Pseudorabies virus (PRV) were propagated in specific-pathogen-free (SPF) chicken embryos or MDCK cells as previously described (37). Muscovy Duck Reovirus (MDRV) and herpes simplex virus type 1 (HSV-1) were kindly provided by Prof. Jinghua Yan (Institute of Microbiology, Chinese Academy of Sciences, Beijing, China) and propagated in Vero cells (38).

### Antibodies and Other Reagents

The following antibodies were used in this study: phospho-STAT1 (Tyr701), ISG15, Mx1, IFITM3, phospho-IκBα (Ser32), IκBα, phospho-NF-κB p65 (Ser536), NF-κB p65 (Cell Signaling Technolohgy), ILF2, ILF3, phospho-IRF3 (S386) (abcam), and β-actin (Santa Cruz Biotechnology). Low molecular weight poly (I:C) and BAY 11-7082 were purchased from InvivoGen and Santa Cruz Biotechnology separately.

### Cell Culture

A549 (human type II alveolar epithelial cells), 293T (human embryonic kidney cells), K562 (human chronic myelogenous leukemia cells), MCF7 (human breast adenocarcinoma cells), NIH/3T3 (mouse embryonic fibroblast cells), 4T1 (mouse mammary tumor cells) and MDCK (Madin-Darby canine kidney cells) were purchased from American Type Culture Collection (ATCC, Manassas, VA, USA), while Huh7 (human hepatocellular carcinoma cells), HeLa (human cervical cancer cells) and LLC (mouse Lewis lung carcinoma cells) were from the National Infrastructure of Cell Line Resource (NICR, Beijing, China). All cell lines were maintained in Dulbecco’s Modified Eagle’s Medium (DMEM) or RPMI1640 (Gibco-BRL, Inc., Gaithersburg, MD, USA) containing 10% fetal calf serum (FCS) supplemented with penicillin (100 U/ml) and streptomycin (100 μg/ml).

### RNA Fluorescence *In Situ* Hybridization (RNA-FISH)

Human RDUR (lnc1101049), U6 (lnc110101) and 18sRNA (lnc110102) probes were synthesized by RiboBio Company (Guangzhou, China). RNA-FISH was performed according to the protocol (39). The slides were stained with DAPI and the fluorescent signal was detected under a Leica SP8 confocal laser microscope (Wetzlar, Germany).

### 5′ and 3′ RACE

The 5′ and 3′ RACE assays were performed using the SMARTer RACE cDNA amplification Kit (Clontech, Madison, USA) following the manufacturer’s instructions.

### Mouse Experiment

The RDUR-deficient mice were generated by CRISPR/Cas9 as described previously (40). RIG-I knockout mice were kindly provided by Prof. Zhugang Wang (Shanghai Jiao Tong University, Shanghai, China) (41). For infection, mice were inoculated intranasally with 5×10^4^ PFU of the A/WSN/33 virus. At indicated time post-infection (p.i.), mice were euthanized and the organs were removed aseptically for further analysis.

### Histopathological Analysis

Mouse tissues were fixed in 4% paraformaldehyde and embedded in paraffin. Then 4-mm-thick sections were prepared and stained with H&E. The slides were visualized under an Olympus BH-2 microscope (Tokyo, Japan).

### Cell Extracts and Western Blotting

Preparation of cell extracts and Western blotting were performed as previously described (42). Briefly, cell lysates were separated by SDS-polyacrylamide gel electrophoresis and transferred onto a nitrocellulose membrane, and probed with indicated antibodies as described previously (43).

### HA Assay and Plaque Assay

Hemagglutinin (HA) assay and plaque assay were performed as previously described (37). To determine virus titers in lungs, infected tissues were homogenized and centrifuged at 2,000 × *g* for 10 min, and the supernatants were titrated by plaque assay as described above (37).

### RNA Preparation, RT-PCR and Quantitative Real-Time PCR (qRT-PCR)

Total RNA was extracted from cells or tissues and cDNA was synthesized, and RT-PCR and qRT-PCR were performed as previously described (30). β-actin or GAPDH was chosen as a reference gene.

### ELISA Assay

Wide type (WT) mice and RDUR knockout mice were infected with IAV. Lung tissues were lysed and cytokines were quantified by ELISA kits following the manufacturers’ instructions. Mouse IFN-β (Cat. No. 439407), mouse IL-1β (Cat. No. ab100704) and mouse IL-6 (Cat. No. BMS603) ELISA kits were purchased from BioLegend, Abcam and Invitrogen, respectively.

### RNA Pull-Down and RNA Immunoprecipitation (RIP) Assay

RNA was *in vitro* transcribed using MEGAscript™ T7 Transcription Kit (Cat. No. AM1333, Invitrogen) and biotinylated using Pierce RNA 3’-End Desthiobiotinylation Kit (Cat. No. 20163, Pierce) following the kits’ instructions. A549 cell lysates were used for RNA pull-down assay using Pierce Magnetic RNA-Protein Pull-Down Kit according to the manufacturer’s protocol (Cat. No. 20164, Pierce). The mixture of retrieved proteins was detected by mass spectrometry. RIP assay was performed using the Magna RIP™ RNA-Binding Protein Immunoprecipitation Kit (Cat. No. 17-701, Millipore) following the manufacturer’s instruction.

### Statistical Analysis

Comparison between groups was made using Student’s *t*-test. Data represent the mean ± standard deviation (SD). Differences were considered statistically significant with *P*<0.05.

## Results

### Human RDUR Is Identified as a New lncRNA Induced by Viral Infection

**Figure 1:**
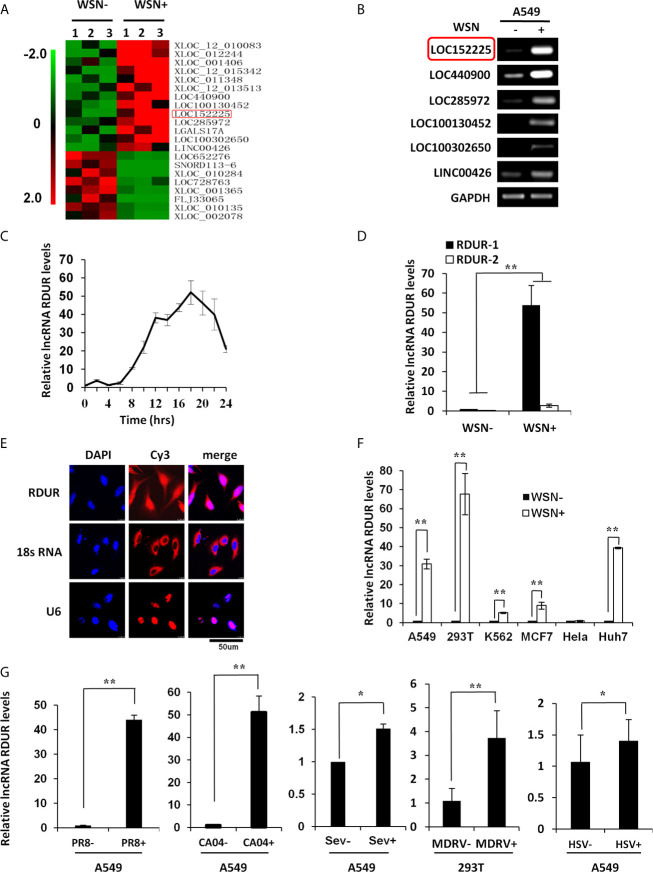
Human RDUR is identified as a new lncRNA induced by influenza A virus and several other viruses. **(A)** The differentially expressed lncRNAs in A549 cells infected with or without A/WSN/33 influenza virus (WSN) were analyzed by a cDNA microarray (http://www.ncbi.nlm.nih.gov/geo/; GEO access number GSE58741). Shown are representative differentially expressed lncRNAs. **(B)** A549 cells infected with WSN were collected at 16 h post-infection (hpi). The differential expressions of 6 selected lncRNAs were confirmed by RT-PCR. RDUR (LOC152225) is indicated by red rectangle. **(C)** Quantitative real-time PCR (qRT-PCR) was performed to examine the kinetics of RDUR expression in WSN infected A549 cells (n = 3; means ± SD). **(D)** Shown is the abundance of two isoforms of RDUR examined by qRT-PCR. The error bars represent the SD. Shown are representative results from three independent experiments. **(E)** RNA-FISH was performed to determine the localization of RDUR in WSN infected A549 cells. Shown were representative images from at least three independent experiments with similar results. **(F)** RDUR expression was examined by RT-PCR in indicated human cell lines infected with WSN (moi=1) for 16 h. Plotted are the average results from three independent experiments. Data are shown as means ± SD. **P < 0.01. **(G)** RDUR expression was examined by qRT-PCR in indicated cells infected with influenza viruses (PR8 and CA04), Sendai virus (SeV), Muscovy duck reovirus (MDRV), or herpes simplex virus type 1 (HSV-1). Plotted are the average results from three independent experiments. Data are shown as means ± SD. *P < 0.05, **P < 0.01.

Our previous study has identified lncRNA NRAV as a key regulator of initial transcription of multiple critical ISGs (30), indicating the functional involvement of lncRNAs in innate immunity against viral infection. To gain more insights into the roles of lncRNAs in the pathogenesis of IAV, a new genome-wide lncRNA microarray was employed to analyze differentially expressed lncRNAs in human alveolar epithelial cells (A549) infected with A/WSN/33 (H1N1) influenza virus. Gene Expression Omnibus (GEO) accession number for the microarray is GSE58741, and representative differentially expressed lncRNAs were clustered (**Figure 1A**). To identify the functional lncRNAs associated with IAV infection, six most significantly upregulated lncRNAs were selected as candidates for further study. As shown in **Figure 1B**, increased expression of these selected lncRNAs was confirmed by RT-PCR. Since RIG-I is a main sensor involved in the induction of innate immunity against IAV (44), we performed screening by using RIG-I knockdown A549 cells and found that silencing RIG-I caused a marked decrease in the expression of lncRNA LOC152225 as compared to the control group following IAV infection (**Supplementary Figure S1A**). This lncRNA (LOC152225, also named as LINC02085), here we called RIG-I-dependent IAV-upregulated noncoding RNA (RDUR), was significantly induced by IAV in a time-dependent manner (**Figure 1C**). Thus, RDUR was chosen for in-depth study. The human lncRNA gene *rdur* is located on chromosome 3q12.3 and its upstream gene is NFKBIZ, ˜137Kb from *rdur*. Determination of 5 and 3 ends of lncRNA RDUR by rapid amplification of cDNA ends (RACE) revealed that RDUR has two transcripts in human (**Supplementary Figure S1B**). Additionally, the abundance of the two transcripts was examined by qRT-PCR and RT-PCR. The results showed that the short isoform (RDUR-1) was a major transcript of RDUR in IAV-infected cells (**Figure 1D** and **Supplementary Figure S1C**). The protein coding potential was analyzed by CPC (http://cpc2.cbi.pku.edu.cn/index.php), CPAC (http://lilab.research.bcm.edu/cpat/index.php) and PORTRAIT (http://bioinformatics.org/portrait/) algorithms. All these analyses indicated no protein-coding potential in RDUR sequences. Indeed, no protein expression was detected in the cells transfected with Flag-tagged RDUR expression vector (**Supplementary Figure S1D**). RNA fluorescence *in situ* hybridization (RNA-FISH) and cell fractionation experiments showed that RDUR was localized both in the cytoplasm and nucleus of A549 cells (**Figure 1E** and **Supplementary Figure S1E**).

In addition, we observed that RDUR was expressed in various human cell lines, and its expression was markedly upregulated in IAV-susceptible cells but not in IAV-insusceptible (HeLa) cells (**Figure 1F** and **Supplementary Figure S1F**), suggesting that robust expression of RDUR is associated with IAV infection. RDUR was also obviously induced by infections with several other viruses, including the negative ssRNA virus Sendai virus (SeV), dsRNA virus Muscovy Duck Reovirus (MDRV), DNA virus herpes simplex virus type 1 (HSV-1) and Pseudorabies virus (PRV), as well as other IAV H1N1 viruses PR8 and CA04 (**Figure 1G** **and** **Supplementary Figures S1G, H** **)**. Collectively, these data indicate that RDUR can be induced by a variety of viruses.

### RDUR Suppresses IAV Replication *In Vitro*

**Figure 2:**
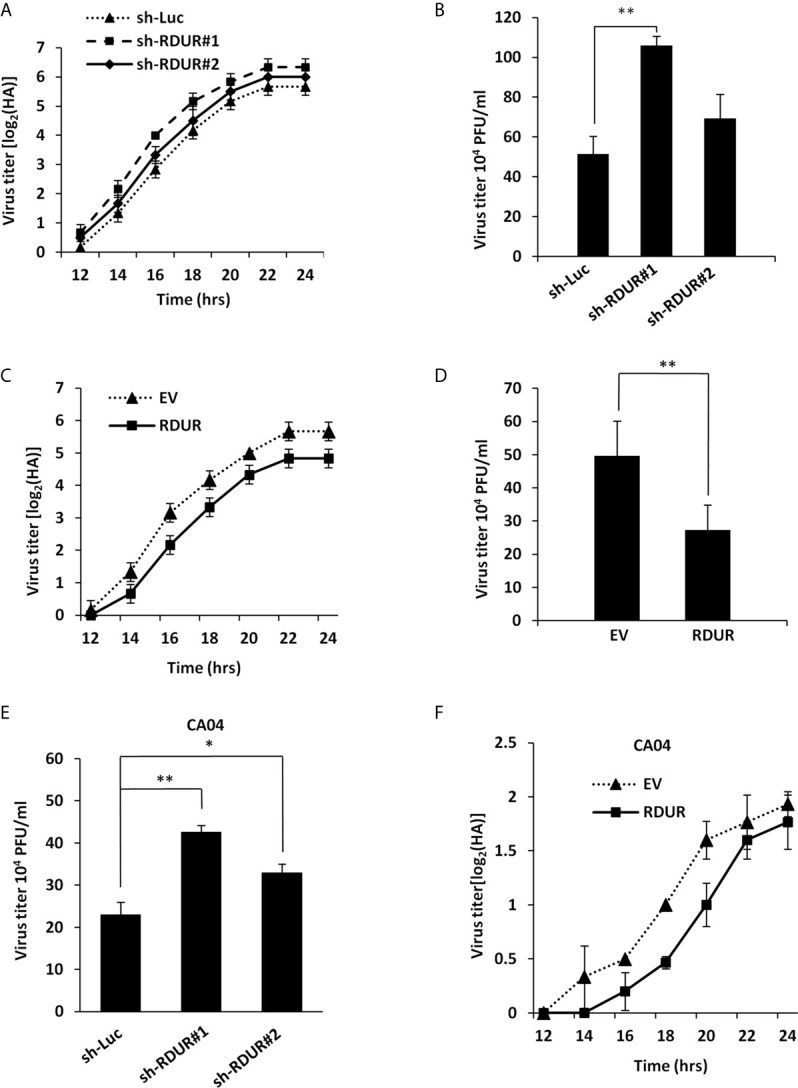
Altering RDUR expression impacts IAV replication in A549 cells. **(A)** RDUR knockdown A549 cell lines were infected with WSN and the virus titers in the supernatants were measured by hemagglutinin (HA) assay at indicated time points. Luciferase shRNA (sh-Luc) served as a control. Shown are average results from three independent experiments. Data are shown as means ± SD. **(B)** RDUR knockdown A549 cells were infected with WSN for 16 h and the virus titers were measured by plaque assay. Plotted are the average levels from three independent experiments. Data are shown as means ± SD, **P < 0.01. **(C)** A549 cells overexpressing RDUR were infected by WSN and the virus titers at indicated time points were measured as described in **(A)**. Data are shown as means ± SD. **(D)** A549 cells overexpressing RDUR were infected by WSN for 16 h and the virus titers were measured by plaque assay. Cells expressing empty vector (EV) were used as control. Plotted are the average results from three independent experiments. Data are shown as means ± SD, **P < 0.01. **(E)** RDUR knockdown A549 cells were infected by CA04 virus for 16 h and the titers were measured by plaque assay. Plotted are the average levels from three independent experiments. Data are shown as means ± SD. *P< 0.05, **P < 0.01. **(F)** RDUR overexpressed A549 cells were infected by CA04 virus and the virus titers were measured by HA assay at indicated time points. Data are shown as means ± SD.

Since IAV induced robust expression of RDUR in host cells, we hypothesized that RDUR might play a critical role in IAV replication. To test this hypothesis, we generated A549 cell lines stably expressing specific shRNAs targeting RDUR using the shRNA-based lentiviral vectors. As shown in **Supplementary Figure 2A**, sh-RDUR#1 exhibited a better efficiency than sh-RDUR#2 in silencing RDUR. Of note, knockdown of RDUR obviously promoted the IAV replication, as evidenced by increased virus titers through hemagglutinin (HA) assay and plaque assay (**Figures 2A, B** **)**. In addition, we observed that sh-RDUR#1 had a more profound effect on the viral replication than sh-RDUR#2. To further determine the functional relevance of RDUR in IAV replication, we generated A549 cell lines stably overexpressing RDUR using the lentiviral overexpression system (**Supplementary Figures S2B, C** **)**. Both HA assay and plaque assay demonstrated that IAV replication was consistently impaired in RDUR-overexpressing A549 cells (**Figures 2C, D** **)**.

Moreover, we examined the effect of RDUR on the replication of other H1N1 influenza virus strains including CA04 and PR8. Similarly, RDUR knockdown increased the replication of these viruses (**Figure 2E** and **Supplementary Figure S2D**), whereas RDUR overexpression impaired the replication of these viruses (**Figure 2F** and **Supplementary Figure S2E**). These data suggest that RDUR suppresses the replication of IAV in host, and the induction of RDUR might be an innate antiviral response required for viral clearance.

### RIG-I Is Required for IAV-Induced Expression of RDUR *In Vitro* and *In Vivo*

**Figure 3:**
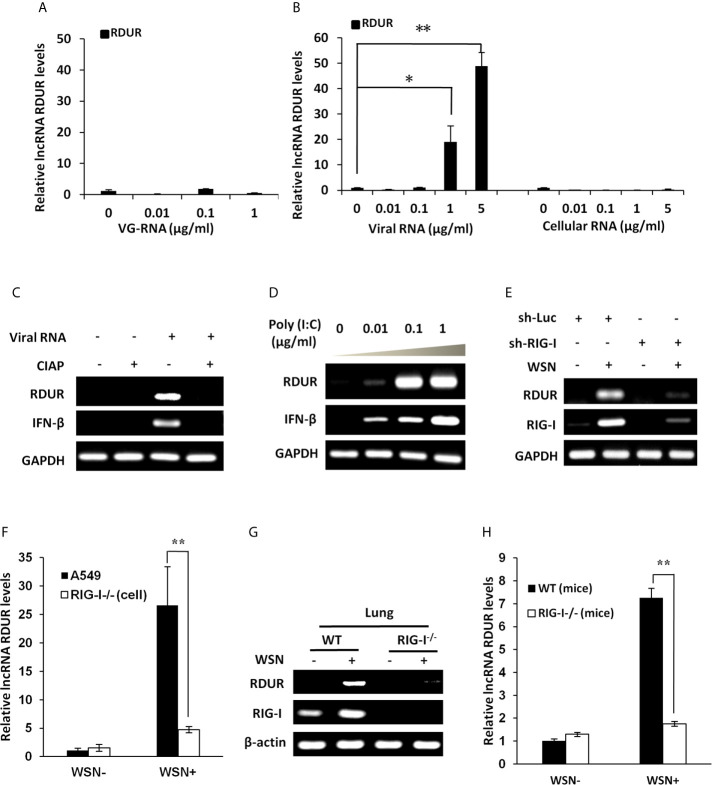
IAV-induced robust expression of RDUR is RIG-I dependent both *in vitro* and *in vivo*. **(A)** A549 cells were transfected with indicated amount of WSN genomic RNA (VG-RNA) using Lipofectamine 2000. Effect of VG-RNA on the expression of RDUR was determined by qRT-PCR. **(B)** Different amounts of total RNA from A549 cells infected with (named as Viral RNA) or without (named as Cellular RNA) WSN virus were transfected into native A549 cells respectively using Lipofectamine 2000. Expression of RDUR in transfected A549 cells was examined by qRT-PCR. **(C)** Viral RNA from IAV-infected A549 cells was treated with/without alkaline phosphatase calf intestinal (CIAP), and was transfected into native A549 cells. Expression of RDUR in A549 cells was then examined by RT-PCR. **(D)** Different amounts of poly (I:C) were transfected into A549 cells and expression of RDUR in transfected A549 cells was examined by RT-PCR. **(E)** A549 cell line stably expressing shRNAs targeting RIG-I was generated. Then, the cells were infected with WSN for 16 h, and the expression of RDUR was examined by RT-PCR. **(F)** RIG-I knockout A549 cells were generated by utilizing CRISPR-Cas9. These cells and native A549 cells were infected with WSN for 16 h, and the expression of RDUR was examined by qRT-PCR. **(G, H)** RIG-I knockout mice were infected with WSN for 1 day, and the expression of RDUR in lungs was detected by RT-PCR **(G)** and qRT-PCR **(H)**. Shown are representative data from three independent experiments. The error bars represent the SD, *P < 0.05, **P < 0.01.

Next, we were interested in identifying how RDUR is induced during IAV infection. Firstly, we investigated whether viral RNA is responsible for the induction of RDUR. For this, A549 cells were treated with different doses of genomic RNA (VG-RNA) directly isolated from WSN virus (**Figure 3A** and **Supplementary Figure S3A**) or total RNA derived from IAV-infected (viral RNA) or uninfected (cellular RNA) cells (**Figure 3B** and **Supplementary Figures S3B, C** **)**. The results showed that only viral RNA isolated from IAV-infected cells but not the VG-RNA or cellular RNA was able to upregulate the expression of RDUR.

It is known that viral RNA produced during IAV replication contains 5’-triphosphorylated RNA that is the ligand for RIG-I, which is critical for the activation of innate immune signaling (45–47). Next, we utilized calf intestine alkaline phosphatase (CIAP) to remove the 5’-triphosphate terminus of the viral RNA. As expected, viral RNA after CIAP treatment lost the ability to trigger the expression of RDUR (**Figure 3C**). In addition, treatment with low molecular weight Poly (I:C), a RIG-I agonist, dramatically induced the expression of RDUR in A549 (**Figure 3D**). The above observations suggest that induction of RDUR in IAV-infected cells may be regulated by RIG-I.

RIG-I is an important PRR belonging to RLR family that includes key cytoplasmic pathogen recognition receptors involved in detection of various viruses. To further substantiate whether RLRs are involved in the regulation of RDUR, A549 cells were infected with lentiviral mediated shRNAs specifically targeting either RIG-I or MDA5. The results revealed that knockdown of RIG-I, but not MDA5 markedly suppressed IAV-induced expression of RDUR (**Figure 3E** and **Supplementary Figure S3D**). To verify this observation, we further generated RIG-I knockout A549 cell line (**Supplementary Figure S3E**), and found that knockout of RIG-I resulted in a significant decrease in RDUR expression in response to IAV infection (**Figure 3F**).

We also determined whether induction of RDUR by IAV is RIG-I dependent *in vivo*. RDUR homolog sequences in mouse were identified through NCBI Blast in silico analysis. The mouse RDUR (mRDUR) is located on chromosome 16 and its upstream gene is also NFKBIZ. RIG-I knockout mice were then employed and the experiments demonstrated that RIG-I deficiency dramatically suppressed IAV-induced expression of mRDUR in both mouse lungs and embryonic fibroblast cells (MEFs) (**Figures 3G, H** and **Supplementary Figures S3F–H**). Collectively, these results indicate that RIG-I is required for the robust expression of RDUR during IAV infection.

### IAV-Induced RDUR Expression Is Regulated by NF-κB but Not IRF3 and IFNs

**Figure 4:**
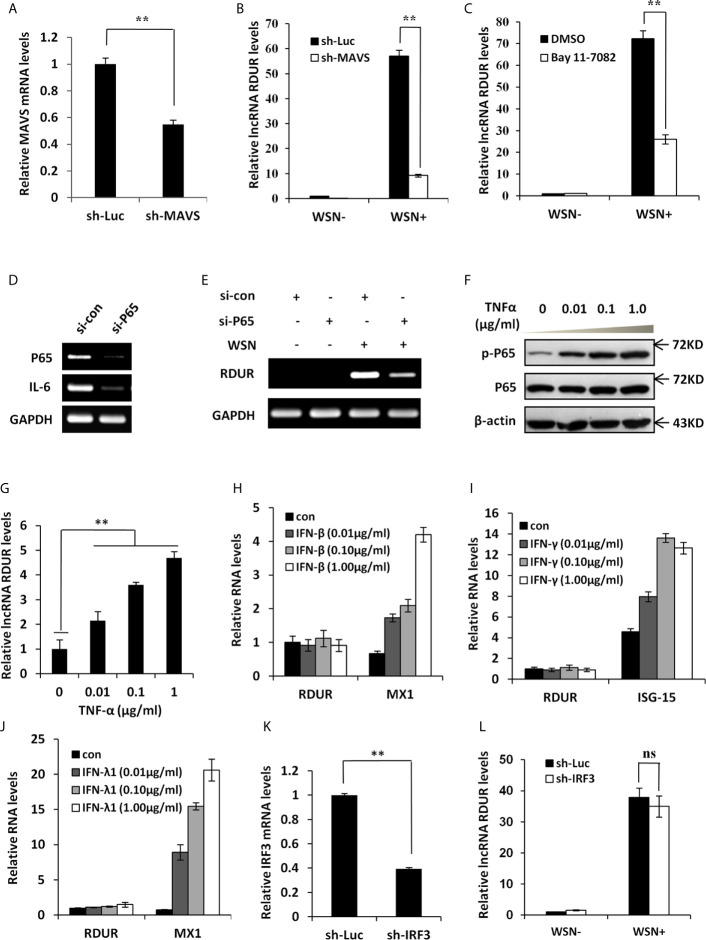
IAV-induced RDUR expression is regulated by NF-κB but not IRF3 and IFNs. **(A)** sh-MAVS and sh-Luc (control) infected A549 cells were analyzed by qRT-PCR to determine the interference efficiency. **(B)** The RNA levels of RDUR in sh-MAVS and sh-Luc (control) A549 cells infected with or without WSN were determined by qRT-PCR. **(C)** The RNA levels of RDUR in A549 cells treated with or without Bay 11-7082 and infected with or without WSN were determined by qRT-PCR. **(D, E)** A549 cells were transfected with siRNAs targeting NF-κB (P65/NFKB3) or control siRNA for 36 h and infected with WSN for 16 h. The knockdown efficiency **(D)** and the RNA level of RDUR **(E)** were then detected by RT-PCR. **(F, G)** A549 cells were stimulated with TNF-α for 180 min, and the activation of NF-κB was confirmed by Western blotting **(F)** and the expression of RDUR was examined by qRT-PCR **(G)**. **(H, J)** A549 cells were treated with different amounts of IFNs including IFN-β **(H)**, IFN-γ **(I)** and IFN-λ1 **(J)** for 90 min. The expression levels of RDUR and ISGs (Mx1 or ISG15) in the cells were examined by qRT-PCR. **(K)** Shown is IRF3 (sh-IRF3) knockdown efficiency analyzed by qRT-PCR in A549 cells. **(L)** The RNA levels of RDUR in sh-IRF3 and sh-Luc A549 cells infected with or without WSN were determined by qRT-PCR. Shown are representative data from three independent experiments. Plotted are the average levels from three independent experiments. The error bars represent the SD, **P < 0.01, and ns represents no statistical significance.

Because RIG-I interacts with MAVS to regulate the downstream signaling pathway, we generated A549 cell line stably expressing shRNA targeting MAVS to determine the effect of MAVS on RDUR expression (**Figure 4A**). The results showed that IAV-induced expression of RDUR was significantly suppressed by MAVS knockdown (**Figure 4B**), supporting that the expression of RDUR is regulated by the RIG-I/MAVS signaling pathway. Since NF-κB is a key transcriptional factor downstream of the RIG-I/MAVS pathway (48), we next examined whether NF-κB is implicated in the regulation of RDUR expression during IAV infection. For this, NF-κB inhibitor Bay 11-7082 was used. The results showed that IAV-induced expression of RDUR was significantly attenuated in A549 cells treated with Bay 11-7082 (**Figure 4C** and **Supplementary Figure S4A**). Because Bay 11-7082 is not a specific inhibitor of NF-κB, we attempted to provide more supportive evidence by using siRNA to knockdown NF-κB. Supportively, silencing NF-κB p65 subunit by siRNA also markedly suppressed IAV-induced expression of RDUR (**Figures 4D, E** **)**. In contrast, NF-κB activators such as TNF-α and IL-1β enhanced the expression of RDUR in A549 cells (**Figures 4F, G** and **Supplementary Figures S4B, C** **)**. These data suggest that upregulation of RDUR is regulated by the NF-κB signaling pathway in IAV infected cells.

To examine whether IFNs could stimulate the expression of RDUR, A549 cells were treated with different doses of IFNs such as IFN-β, IFN-γ, and IFN-λ1. Clearly, these IFNs failed to induce the expression of RDUR (**Figures 4H–J** and **Supplementary Figures S4D–F**). IRF3 is another transcription factor downstream of RIG-I signaling, which plays a vital role in innate immunity against viral infection (49). The above findings prompted us to examine whether IAV-induced expression of RDUR is mediated by IRF3. Disrupting IRF3 had no significant effect on RDUR expression after WSN infection (**Figures 4K, L** and **Supplementary Figure S4G**). These results reveal that NF-κB, but not IRF3 and IFNs, participates in the induction of RDUR. Taken together, our findings suggest that RDUR expression is regulated through the RIG-I/MAVS/NF-κB signaling pathway during IAV infection.

### mRDUR Knockout Mice Are More Susceptible to IAV or PRV Infection

**Figure 5:**
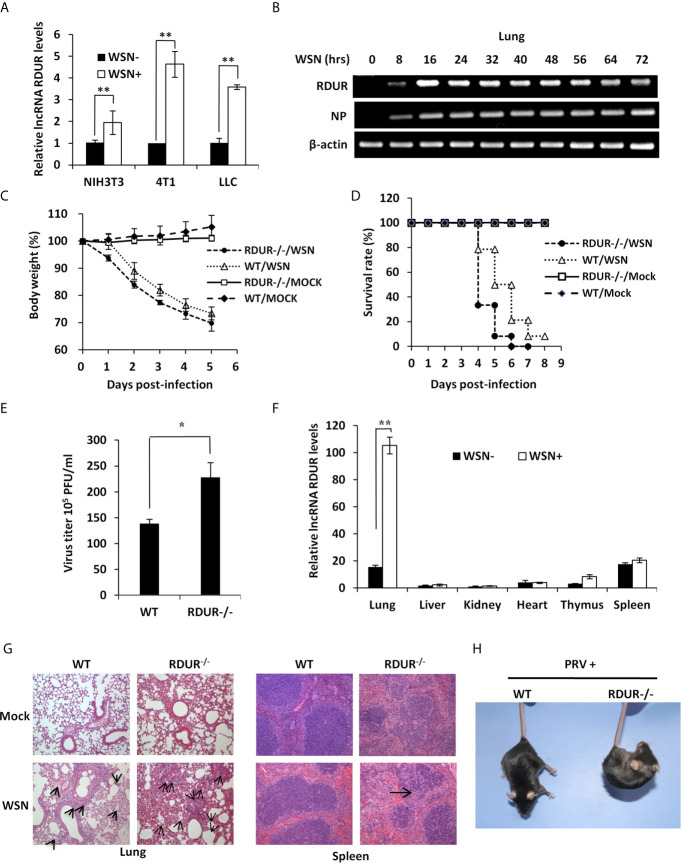
mRDUR-deficient mice are more susceptible to IAV and PRV infections. **(A)** The expression of mRDUR in three mouse cell lines infected with or without WSN for 16 h was examined by qRT-PCR. The error bars represent the SD, **P < 0.01, *P < 0.05. **(B)** The mRDUR expression levels in the lungs of mice infected with or without WSN for 0-72 h were examined by RT-PCR. **(C, D)** Shown are the body weight change and survival rates of mRDUR knockout and control mice intranasally inoculated with WSN or Mock. Body weight was measured every day (8 mice for each Mock group and 14 mice for each WSN infection group). **(E)** Viral titers in the lungs of mRDUR knockout and control mice inoculated intranasally with WSN for 72 h were measured by plaque assay. Plotted are the average levels from three independent experiments. The error bars represent the SD, *P < 0.05. **(F)** Expression levels of mRDUR in different organs (lung, liver, kidney, heart, thymus and spleen) of mice infected with WSN or Mock were examined by qRT-PCR (n = 3; means ± SD; **P < 0.01). **(G)** Shown are representative micrographs of H&E stained lung (left) and spleen (right) sections of the mRDUR knockout and control mice infected with or without WSN. **(H)** WT and RUUR-/- mice were intramuscularly infected with PRV for 2 days, and shown is representative clinic-pathological phenotype from three independent experiments.

To define the functional relevance of RDUR in IAV pathogenesis, we sought to develop a mouse model for *in vivo* studies. For this, firstly we tested whether mRDUR could be induced by IAV in mouse cells. Indeed, IAV infection also greatly induced the expression of mRDUR in mouse cell lines (**Figure 5A** and **Supplementary Figure S5A**). Next, we examined whether IAV infection can induce the expression of mRDUR in mice. Our time course experiments showed that the expression of mRDUR could be consistently induced by several IAV strains including WSN, PR8 and CA04 viruses (**Figure 5B** and **Supplementary Figures S5B, C** **)**. These results provide strong evidence that IAV infection triggers mRDUR expression both *in vitro* and *in vivo*.

To test the functional involvement of mRDUR in IAV pathogenesis, we next generated mRDUR knockout mice using CRISPR-Cas9 (**Supplementary Figures S5D, E** **)**. The mRDUR knockout mice lost body weight faster and died earlier, compared to the WT littermates after IAV infection (**Figures 5C, D** **)**. Infected mRDUR-deficient mice exhibited a consistent decrease in body weight, with an average loss of about 25% on 4 days post-infection (dpi). On 5 dpi, only approximately 8% of infected mRDUR-deficient mice survived, whereas nearly 50% of WT mice remained alive (**Figure 5D**). On 6 dpi, no mRDUR knockout mice survived, while around 21% of WT mice remained alive, indicating that mRDUR knockout mice are more susceptible to IAV challenge than the WT mice. In line with these observations, the plaque assay showed that mRDUR-deficient mice had significantly more viral production in the lungs than the WT animals (**Figure 5E**), suggesting that knockout of mRDUR promotes the IAV replication *in vivo*.

We also tested the expression of mRDUR in various organs including lung, liver, kidney, heart, thymus and spleen of WT mice infected with WSN. The levels of mRDUR in these organs were low upon mock infection, but a drastic upregulation of mRDUR was observed in the lungs after infection with IAV (**Figure 5F**). Importantly, mRDUR knockout mice exhibited a higher degree of acute lung injury and spleen enlargement compared to the WT animals (**Supplementary Figure S5F**). To further evaluate the *in vivo* effect of mRDUR deficiency on IAV pathogenesis, we performed pathologic examination by H&E staining. The results showed more edema and infiltration of inflammatory cells across the interalveolar septum in the lungs, and reduced level of splenic white pulp lymphocytes in spleens of mRDUR knockout mice compared with the WT animals, in response to IAV infection (**Figure 5G**). Together, these data indicate that knocking out of mRDUR in mice increases the susceptibility of the animals to IAV infection.

Additionally, the levels of mRDUR in the lungs and brains were also markedly elevated in the WT mice infected with PRV virus (**Supplementary Figure S5G**). Similarly, mRDUR knockout mice were more susceptible to PRV infection, as evidenced by more severe clinical symptoms than WT littermates (**Figure 5H**). The expression levels of PRV gE mRNA in the lungs were significantly higher in mRDUR knockout mice than in the WT animals (**Supplementary Figure S5H**).

### RDUR Regulates the Expression of Several Critical Antiviral Genes *In Vitro* and *In Vivo*

**Figure 6:**
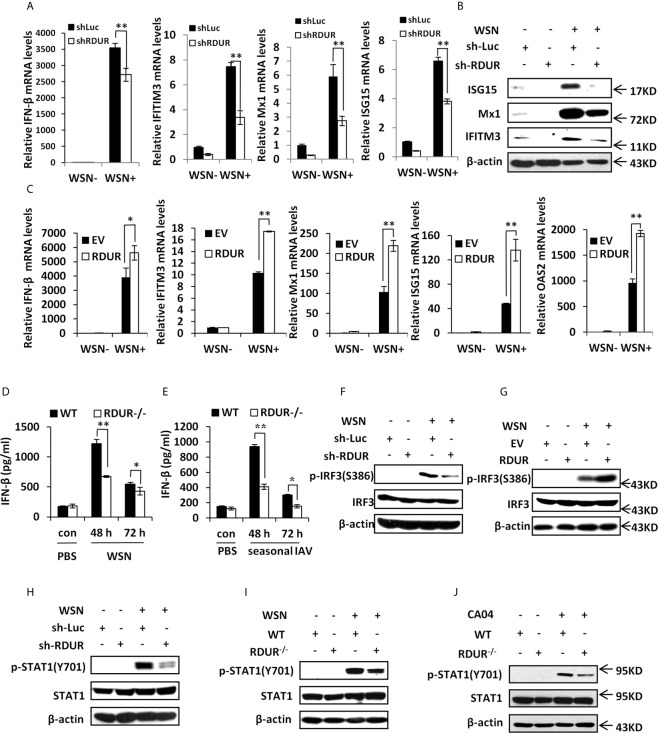
RDUR regulates the expression of several critical antiviral genes *in vitro* and *in vivo*. **(A)** The levels of IFN-β, IFITM3, Mx1 and ISG15 were examined by qRT-PCR in A549 cells expressing shRNAs specifically targeting RDUR or luciferase (control) after infection with or without WSN. Plotted are the average levels from three independent experiments. Data are shown as means ± SD. **P < 0.01. **(B)** A549 cell lines stably expressing specific shRNAs targeting RDUR and luciferase (control) were infected with or without WSN and harvested at 16 hpi, followed by Western blotting with the indicated antibodies. Shown are representative data from three independent experiments with similar results. **(C)** The mRNA levels of IFN-β, IFITM3, Mx1, ISG15 and OAS2 in RDUR over-expression and empty vector (EV) control A549 cells infected with or without WSN were determined by qRT-PCR. Plotted are the average levels from three independent experiments. Data are shown as means ± SD. *P < 0.05, **P < 0.01. **(D, E)** The protein levels of IFN-β were determined by ELISA in the lungs of mRDUR knockout or WT mice infected with WSN or seasonal IAV (3 mice each group). Plotted are the average levels from three independent experiments. Data are shown as means ± SD. *P < 0.05, **P < 0.01. **(F)** RDUR knockdown and control A549 cells were infected with or without WSN, and Western blotting was performed to detect indicated proteins. **(G)** RDUR overexpression and EV control A549 cells were infected with or without WSN and examined by Western blotting with the indicated antibodies. **(H)** A549 cell lines stably expressing specific shRNAs targeting RDUR and luciferase (control) were infected with or without WSN and examined by Western blotting with the indicated antibodies. **(I, J)** mRDUR knockout and control mice were infected with WSN or CA04 influenza virus. Western blotting was performed by using indicated antibodies. Shown are representative immunoblots from three independent experiments.

In an attempt to define the mechanism underlying the involvement of RDUR in IAV replication and pathogenesis, we determined whether RDUR had any effects on the expression of antiviral genes such as IFNs and ISGs. The results showed that knockdown of RDUR downregulated the mRNA levels of IFN-β and some critical ISGs including IFITM3, Mx1 and ISG15 in A549 cells infected with WSN (**Figure 6A** and **Supplementary Figure S6A**). This was further confirmed by Western blotting and ELISA (**Figure 6B** and **Supplementary Figure S6B**) for the detection the protein levels of these ISGs and IFN-β. By contrast, overexpression of RDUR upregulated the levels of IFN-β and ISGs in the cells infected with WSN (**Figure 6C** and **Supplementary Figure S6C**). We further validated the findings in mRDUR knockout and WT mice challenged with IAV. Consistently, mRDUR knockout mice exhibited significantly lower mRNA levels of IFNs and ISGs in the lungs, compared to the WT animals infected with WSN **(** **Supplementary Figure S6D**). Moreover, as detected by ELISA, mRDUR knockout mice also showed significantly lower protein level of IFN-β in the lungs, compared to the WT controls infected with WSN (**Figure 6D**). Similar data were obtained from the mice infected with a seasonal H1N1 influenza virus (**Figure 6E**). These results indicate that RDUR functions as a positive regulator of IFNs and ISGs during IAV infection both *in vitro* and *in vivo*.

Since IRF3 is a well known transcription factor governing expression of IFNs in innate immune response to viral infection, we next examined the influence of RDUR on the phosphorylation of IRF3 (p-IRF3) on Ser386. Upon IAV infection, p-IRF3 was obviously impaired in RDUR knockdown cells (**Figure 6F**). On the contrary, p-IRF3 level increased in RDUR overexpressing cells after IAV infection (**Figure 6G**). These observations suggest that RDUR promotes the expression of IFNs possibly by regulating IRF3 activation.

Virus-induced expression of various critical ISGs is mediated by cytokine-activated JAK/STAT signaling. Next, we determined whether RDUR has any effects on the activation of JAK/STAT signaling. To this end, phosphorylation of STAT1 (p-STAT1) on Tyr701 was examined by Western blotting. As predicted, knockdown of RDUR profoundly attenuated WSN-induced p-STAT1 in A549 cells (**Figure 6H**). Similarly, knockout of mRDUR also remarkably weakened WSN-induced p-STAT1 in the lungs of mice (**Figure 6I**). The finding was further confirmed by experiments employing CA04 and seasonal H1N1 viruses (**Figure 6J** and **Supplementary Figure S6E**). Together, the results suggest that RDUR enhances host antiviral immunity by positively regulating the IRF3/IFNs axis, thereby activating JAK/STAT1 signaling that triggers the expression of ISGs.

### RDUR Deficiency Activates NF-κB and Enhances Inflammatory Response After IAV Infection

**Figure 7:**
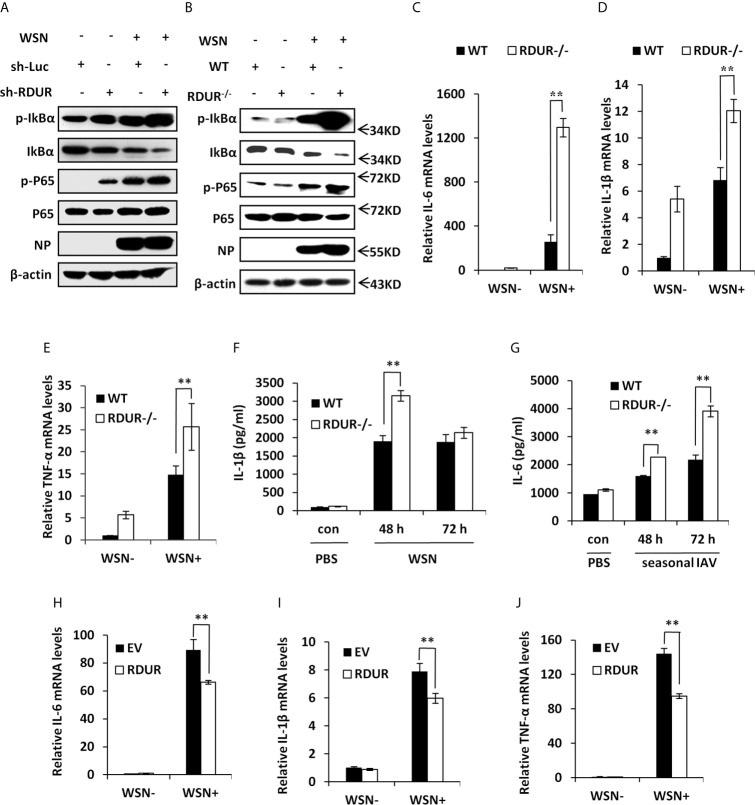
RDUR deficiency induces NF-κB activation and aggravates inflammatory response after IAV infection *in vivo*. **(A)** A549 cell lines stably expressing specific shRNAs targeting RDUR and luciferase (control) were infected with or without WSN and harvested at 16 hpi., followed by Western blotting with the indicated antibodies. **(B)** Shown are the protein levels in the lungs of mRDUR knockout and WT mice infected with WSN or Mock, detected by Western blotting with the indicated antibodies. **(C–E)** The mRNA levels of IL-6 **(C)**, IL-1β **(D)** and TNF-α **(E)** in the lungs of mRDUR knockout and WT mice infected with WSN or Mock were determined by qRT-PCR. Plotted are the average results from three independent experiments. Data are shown as means ± SD. **P < 0.01. **(F, G)** The Protein levels of IL-1β **(F)** and IL-6 **(G)** in the lungs of mRDUR knockout and WT mice infected with WSN and seasonal IAV or Mock were determined by qRT-PCR (3 mice per group). Plotted are the average results from three independent experiments. Data are shown as means ± SD. **P < 0.01. **(H–J)** The mRNA levels of IL-6 **(H)**, IL-1β **(I)** and TNF-α **(J)** in RDUR overexpression and EV control A549 cells infected with WSN or Mock were determined by qRT-PCR. Plotted are the average results from three independent experiments. Data are shown as means ± SD. **P < 0.01.

NF-κB is a key transcriptional factor downstream of RIG-I pathway and involved in many cellular processes, especially inflammation (16). Having observed that RDUR deficiency caused severe inflammation in the lungs of infected mice, we next investigated the effect of RDUR on NF-κB activation. Surprisingly, knockdown of RDUR resulted in a dramatic increase in the level of both IκBα phosphorylation (Ser32) and NF-κB p65 phosphorylation (Ser536) during the IAV infection (**Figure 7A**). In line with this, knockout of mRDUR also resulted in a stronger activation of NF-κB in the lungs of mice infected with IAV (**Figure 7B**). These results indicate that RDUR deficiency enhances IAV-induced activation of NF-κB *in vitro* and *in vivo*.

In addition, we noticed that the mRNA levels of pro-inflammatory cytokines IL-6, IL-1β and TNF-α were significantly increased in the lungs of mRDUR knockout mice compared to the WT animals infected with IAV (**Figures 7C–E**). Knockout of mRDUR also significantly increased the protein levels of IL-1β and IL6 in the lungs of mice infected with either WSN or seasonal H1N1 influenza virus (**Figures 7F, G** **)**. The increased levels of these pro-inflammatory cytokines in the lungs of infected mRDUR knockout mice indicate that mRDUR might be responsible for tight control of inflammatory responses during virus infection. To verify these findings, RDUR overexpressing A549 cell lines were used to examine the effect of RDUR on the expression of pro-inflammatory cytokines. As expected, the expression of these pro-inflammatory cytokines was significantly suppressed in RDUR overexpressing cells (**Figures 7H–J**). Together, these results suggest that the RDUR can counter virus infection by positively regulating the expression of IFNs and their stimulating genes, and meanwhile alleviate harmful inflammatory response *via* a negative feedback control of NF-κB activation during IAV infection.

### RDUR Interacts With ILF2 and ILF3 RNA Binding Proteins

**Figure 8:**
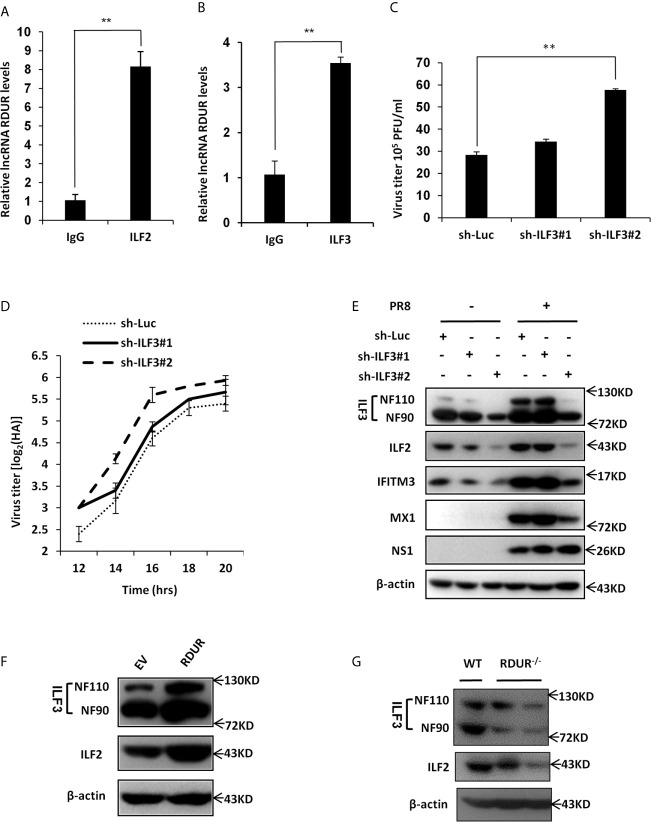
RDUR interacts with ILF2 and ILF3 RNA binding proteins. **(A, B)** RIP assay was performed using ILF2-specific antibody **(A)** or ILF3-specific antibody **(B)** or normal IgG control and quantitative real-time PCR was performed to detect RDUR in A549 cells infected with IAV for 16 h. **(C)** ILF3 knockdown A549 cells were infected with IAV for 14 h and the virus titers were measured by plaque assay. Plotted are the average levels from three independent experiments. Data are shown as means ± SD, **P < 0.01. **(D)** ILF3 knockdown A549 cell lines were infected with PR8 and the virus titers in the supernatants were measured by HA assay at indicated time points. Shown are average results from three independent experiments. Data are shown as means ± SD. **(E)** A549 cell lines stably expressing specific shRNAs targeting ILF3 and luciferase (control) were infected with or without IAV and harvested at 14 hpi., followed by Western blotting with the indicated antibodies. **(F)** A549 cells overexpressing RDUR were lysed and Western blotting was performed to detect indicated protein levels. **(G)** The ILF2 and ILF3 level in the lungs of WT and mRDUR knockout mice were detected by Western blotting.

Many lncRNAs can physically bind to RNA binding proteins to regulate their functions (50–52). To identify RDUR binding proteins, we performed an RNA pull-down assay using biotinylated RDUR. Biotinylated sense RDUR and antisense RDUR (as a control) were incubated with total protein extract of A549 cell lysates and pulled down with streptavidin magnetic beads. The mixture of retrieved proteins was then analyzed by mass spectrometry. Interestingly, ILF2 and ILF3 (also known as NF45 and NF90/NF110 or NFAR) RNA binding proteins were identified as RDUR interactors with relatively high scores (**Supplementary Table 1**). This finding was further confirmed by RNA immunoprecipitation (RIP) with indicated antibodies (**Figures 8A, B**).

ILF2 and ILF3 have been reported to modulate the expression of cellular mRNAs on the post-transcriptional level and function as important host factors in regulating the replication of several RNA viruses, such as HIV, HCV, VSV and IAV (53–55). To investigate the role of ILF3 in the pathogenesis of IAV, ILF3 knockdown A549 cell lines were generated and IAV loads and replication kinetics were examined by plaque and HA assay. Indeed, the virus titer was significantly increased by silencing ILF3 in A549 cells as compared to control (**Figures 8C, D** **)**. Moreover, knockdown of ILF3 resulted in a dramatically decreased expression of some antiviral proteins such as IFITM3 and MX1 and meanwhile activated the NF-κB signaling (**Figure 8E** and **Supplementary Figure S7A**), which was consistent with the observations from RDUR knocking down experiments observed above (**Figures 6**, **7**).

Since it was revealed that ILF2/ILF3 could function through formation of a complex (56), we asked whether RDUR had any effects on the level of ILF2/ILF3 complex. For this, we generated RDUR overexpression cell line and the expression levels of ILF2 and ILF3 were examined. As shown in **Figure 8F**, RDUR overexpression significantly increased the protein levels of ILF2 and ILF3. We further tested the expression level of ILF3 in RDUR knockout mice. As expected, expression levels of both ILF2 and ILF3 were decreased in RDUR knockout mice (**Figure 8G**). We also observed that the protein level of ILF2 was decreased after ILF3 knockdown (**Figure 8E**). This is consistent with previous findings (56). Together, these data suggest that one of mechanisms by which RDUR functions is likely through formation of a complex with ILF2/ILF3 that might serve as an essential regulator of host antiviral responses.

## Discussion

Increasing studies have demonstrated that lncRNAs play important roles in adaptive and innate immunity (19–32). However, the involvement of lncRNAs in innate immune response to viral infection remains largely unknown, and the mechanisms underlying the functions of lncRNAs in these processes are poorly understood. In this study, we identified a new lncRNA termed RDUR that is required for efficient innate immunity against viral infection.

RDUR is expressed in various human and mouse cells and greatly upregulated by infection with different strains of IAV and other viruses, including SeV, MDRV, HSV and PRV. Hence, RDUR-related cellular response might be a universal defense against virus infection. RDUR gene shares high homology between human and mouse, and mRDUR was detectable in multiple organs including lung, spleen and thymus, indicating that RDUR may have broad functions *in vivo*. RDUR is greatly induced in mice by IAV and PRV infection, suggesting its involvement in the viral pathogenesis *in vivo*. Additionally, knockout of mRDUR in mice enhanced the susceptibility of the animals to IAV or PRV infection, suggesting that mRDUR is critical in antiviral response. Therefore, upregulation of RDUR in infected organs/tissues may be initiated by host as a self-protection mechanism, which may be required for efficient viral clearance.

In this study, we demonstrated that RDUR induced by IAV was regulated through the RIG-I-dependent pathway both *in vitro* and *in vivo*. RIG-I is crucial for the recognition of viral RNA in the cytoplasm, and for the production of type I and type III IFNs as well as the activation of downstream signaling pathways in infected cells. NF-κB, as an important regulator of immunity and inflammation, is a key transcriptional factor downstream of the RIG-I pathway (48). Dysregulation of the NF-κB signaling leads to a wide range of disorders ranging from inflammation, autoimmune diseases to oncogenesis (16). Here, we found that induction of RDUR was largely dependent on MAVS and the NF-κB pathway but independent of the IRF3 and IFNs. Together, our experiments demonstrate that the expression of RDUR is mainly regulated through the RIG-I/MAVS/NF-κB pathway. Strikingly, deficiency of mRDUR dramatically increased NF-κB activity and aggravated inflammatory response in IAV-infected mice, indicating that robust expression of mRDUR may prevent the host from severe inflammatory response by opposing IAV-induced NF-κB activation. These data imply that RDUR was upregulated by the RIG-I/MAVS/NF-κB pathway, and in turn, inhibit NF-κB through a negative feedback mechanism during virus infection.

Our results indicate that RDUR could also positively regulate the type I IFNs signaling through regulating transcription factor IRF3. Thus, we propose that RDUR enhances host antiviral immunity by positively activating the IRF3/IFNs axis, thereby activating JAK/STAT signaling that governs the expression of key ISGs. It is worth noting that once the expression of endogenous RDUR was induced by IAV to a certain level, further overexpression of exogenous RDUR apparently could not had more effects on the IRF3/IFNs/STAT axis. The precise mechanism by which RDUR regulates the IRF3/IFN/STAT pathway remains to be determined. In response to viral infection, the induction of RDUR might facilitate virus clearance through a rapid accumulation of the antiviral ISGs. In contrast, knockout of mRDUR increased the susceptibility of mice to IAV infection. This is in good agreement with the concept that a tight and exquisite control of antiviral response ensures a rapid defense against pathogens with minimal inflammatory damage.

Numerous lncRNAs are multi-function molecules and may exert their biological function through interaction with proteins, especially RNA binding proteins (50–52). Here, it was revealed that RDUR could interact with ILF2 and ILF3 RNA binding proteins and positively regulate the levels of ILF2 and ILF3. Recently, ILF3 has been identified as an essential host factor required for the efficient translation of IFN-β1 and a subset of interferon-stimulated genes (55), suggesting an important role for ILF3 in host antiviral defense. One of mechanisms underlying RDUR function is likely through formation of a complex with ILF2/ILF3 that might regulate the translation of IFN-β1 and some ISGs during the viral infection.

In conclusion, we have identified RDUR as a new lncRNA induced by multiple viruses, which inhibits viral replication both *in vivo* and *in vitro*. Robust expression of RDUR triggered by virus is dependent on the RIG-I/NF-κB pathway. Our data suggest that RDUR may be a multi-function lncRNA. On the one hand, it enhances host antiviral immunity by positively activating the IRF3 and upregulating ILF2/ILF3, thereby positively regulating the expression of IFNs and key ISGs. On the other hand, our experiments demonstrate that deletion of RDUR promotes viral infection through downregulating some crucial antiviral genes but activating the NF-κB-dependent inflammatory response, suggesting that virus-induced expression of RDUR may prevent the host from serious inflammation reaction possibly through a mechanism involving a negative feedback control of NF-κB activation and inflammation (**Supplementary Figure S8A**). These findings establish that RDUR, unlike other known lncRNAs such as lncRNA-NEAT1, NRAV and BST2/BISPR, is mainly characterized by its ability to positively regulate interferon-mediated antiviral signaling and balance NF-κB-dependent inflammatory response. Thus, RDUR can thwart virus by increasing expression of IFN-β and ISGs without causing excessive inflammation leading to host tissue injury and organ damage during viral infection. Our studies shed new insights into the complex mechanisms of how host antiviral immunity is regulated by lncRNAs.

## Data Availability Statement

The original contributions presented in the study are included in the article/**Supplementary Material**. Further inquiries can be directed to the corresponding author.

## Ethics Statement

The animal study was reviewed and approved by Research Ethics Committee of Institute of Microbiology, Chinese Academy of Sciences.

## Author Contributions

J-LC, YC, and JH designed the experiments, prepared the figures, and wrote the manuscript. YC, JH, SL, BC, YYL, MX, YL, ZZ, and QP performed the experiments. YC, JO, and J-LC contributed to data analyses. LZ contributed to generation of RDUR-deficient mice. J-LC, KR, and SH contributed to critical comments and revision of the manuscript. All authors contributed to the article and approved the submitted version.

## Funding

This work was supported by National Key Research and Development Program of China (2016YFD0500206), Natural Science Foundation of China (32030110, U1805231 and 31600147), and an open funding project from Key Laboratory of Fujian-Taiwan Animal Pathogen Biology, College of Animal Sciences, Fujian Agriculture and Forestry University.

## Conflict of Interest

The authors declare that the research was conducted in the absence of any commercial or financial relationships that could be construed as a potential conflict of interest.

## Supplementary Material

Supplementary Figure 1 Human RDUR is identified as a new lncRNA whose expression is greatly upregulated by influenza virus infection. **(A)** A549 cells expressing shRNAs targeting RIG-I or luciferase control were infected with or without WSN for 16 h, and the expression of the three lncRNAs was examined by RT-PCR. **(B)** Shown is a paradigm of the genomic location of lncRNA gene RDUR and the relationship with the genes NFKBIZ (light blue) and Zpld1 (light green). **(C)** Shown is the abundance of the two isoforms of RDUR examined by RT-PCR. **(D)** lncRNA RDUR or cDNA of actin (as control) was subcloned into the pFLAG-CMV-5a vector with Flag tag. A549 cell lines stably expressing actin, RDUR or empty vector (EV) were generated and analyzed by Western blotting using Flag antibody. **(E)** A549 cells were fractionated into nuclear and cytoplasmic fractions. RT-PCR was performed to examine the levels of RDUR, nuclear control transcript (U6) and cytoplasmic control transcript (glyceraldehyde 3-phosphate dehydrogenase, GAPDH) in the fractions. T: total RNA, N: nuclear RNA, C: cytoplasmic RNA. Shown are representative results from three independent experiments. **(F)** Human cell lines were infected with or without WSN (moi=1) for 16 h, and RDUR was examined by RT-PCR as described in **Figure 1F**. Shown are representative RT-PCR results from three independent experiments. **(G)** RDUR expression was detected by qRT-PCR in A549 cells infected with PRV virus. Shown are representative data from three independent experiments. **(H)** RDUR expression was examined by RT-PCR in indicated cells infected with different viruses as described in **Figure 1G**. Shown are representative RT-PCR results from three independent experiments. Related to **Figure 1**.

Supplementary Figure 2 Altering RDUR expression impacts IAV replication in A549 cells. **(A)** The efficiency of RDUR knockdown was determined by RT-PCR in control and WSN infected A549 cells. **(B)** The efficiency of RDUR overexpression was determined by qRT-PCR in A549 cells. Plotted are the average levels from three independent experiments. The error bars represent the SD, ***P* < 0.01. **(C)** The efficiency of RDUR overexpression was determined by RT-PCR in control and WSN infected A549 cells. **(D)** RDUR knockdown A549 cells were infected with PR8 for 16 h and the virus titers were measured by plaque assay. Plotted are the average levels from three independent experiments. Data are shown as means ± SD, ***P* < 0.01. **(E)** A549 cells overexpressing RDUR were infected by PR8 virus for 16 h and the titers in the supernatants of the culture medium were measured by plaque assay. Data are shown as means ± SD, ***P* < 0.01. Related to **Figure 2**.

Supplementary Figure 3 IAV-induced robust expression of RDUR is RIG-I dependent both *in vitro* and *in vivo*. **(A)** A549 cells were transfected with indicated amount of WSN genomic RNA (VG-RNA) using Lipofectamine 2000. Effect of VG-RNA on the expression of RDUR and IFN-β was determined by RT-PCR. **(B)** Different amounts of total RNA (named as Viral RNA) isolated from A549 cells infected with the IAV were transfected into native A549 cells using Lipofectamine 2000. The expression levels of RDUR and IFN-β in the transfected A549 cells were examined by RT-PCR. **(C)** Different amounts of total RNA (named as Cellular RNA) from uninfected A549 cells were transfected into native A549 cells using Lipofectamine 2000 and the expression of RDUR and IFN-β in the transfected cells was examined by RT-PCR. **(D)** A549 cell lines stably expressing shRNAs targeting MDA5 was generated. Then, the cells were infected with WSN for 16 h, and the expression of RDUR was examined by RT-PCR. **(E)** RIG-I knockout A549 cell line was generated and examined by Western blotting with indicated antibodies. **(F)** WT and RIG-I knockout mice were sacrificed and the lungs were dissected and lysed, followed by Western blotting to detect the RIG-I level. **(G, H)** MEF cells derived from WT and RIG-I knockout mice were infected with WSN for 16 h, and the expression of RDUR was detected by qRT-PCR (K) and RT-PCR(L), respectively. Shown are representative data from three independent experiments. The error bars represent the SD. ***P* < 0.01. Related to **Figure 3**.

Supplementary Figure 4 IAV-induced RDUR expression is regulated by NF-κB but not IRF3 and IFNs. **(A)** A549 cells were treated with Bay 11-7082 as described in **Figure 4C** and the RNA levels of RDUR were determined by RT-PCR. **(B, C)** A549 cells were stimulated with IL-1β at indicated concentrations for 180 min. The activation of NF-κB was confirmed by Western blotting (B) and the RNA levels of RDUR were examined by qRT-PCR (C). **(D–F)** A549 cells were treated with different IFNs as described in **Figures 4H–J**. The expression levels of RDUR and ISGs (Mx1 or ISG15) were examined by RT-PCR. **(G)** IRF3 knockdown or control cells were infected with or without WSN for 16 h, followed by RT-PCR to detect RDUR levels. Related to **Figure 4**.

Supplementary Figure 5
*In vivo* studies of mRDUR in mice. **(A)** The expression levels of mRDUR in three mouse cell lines infected with WSN for 16 h were examined by RT-PCR. **(B, C)** The expression levels of mRDUR in C57BL/6J mice infected with influenza virus PR8 **(B)** or CA04 **(C)** at indicated time points were examined by RT-PCR. **(D)** Shown is a representative photograph of RDUR knockout C57BL/6J and WT mice used in this study. **(E)** The RDUR knockout mice were generated and genotyped by PCR. Shown is genotyping of RDUR knockout mice: WT, Hetero and Homo represent wild type, heterozygous and homozygous littermates, respectively. Homozygous RDUR knockout mice were indicated by red rectangles. **(F)** mRDUR knockout and control mice were intranasally inoculated with WSN or PBS for 48 h. Then mice were sacrificed and the lungs and spleens were collected. Shown are representative images from three independent experiments. **(G)** WT mice were intramuscularly infected with Pseudorabies virus (PRV) for 2 days, and the mRDUR levels in the lungs and brains of mice were determined by RT-PCR. **(H)** WT and RDUR^-/-^ mice were intramuscularly infected with PRV for 2 days, and the PRV gE mRNA levels were detected in the lungs of mice. Related to **Figure 5**.

Supplementary Figure 6 RDUR regulates the expression of several critical antiviral genes. **(A)** The levels of RDUR, IFN-β, ISG15 and Mx1 in RDUR knockdown A549 cells infected with WSN were determined by RT-PCR. **(B)** Protein levels of IFN-β were determined by ELISA in RDUR knockdown A549 cells. Data are shown as means ± SD. ***P* < 0.01. **(C)** The levels of RDUR, IFN-β, ISG15 and Mx1 in RDUR overexpression A549 cells infected with WSN were determined by RT-PCR. **(D)** The mRNA levels of IFN-β, IL-28A and Mx1 in the lungs of WSN-infected mRDUR knockout or WT mice were determined by qRT-PCR. The error bars represent the SD, ***P* < 0.01. **(E)** mRDUR knockout and control mice were infected with a seasonal H1N1 influenza virus, and Western blotting was performed to detect related proteins. Related to **Figure 6**.

Supplementary Figure 7 RDUR interacts with ILF2 and ILF3 RNA binding proteins. A549 cell lines stably expressing specific shRNAs targeting ILF3 and luciferase (control) were infected with or without IAV and harvested at 14 hpi., followed by Western blotting with the indicated antibodies.

Supplementary Figure 8 The mechanisms of RDUR in antiviral innate immunity. The schematic diagram of RDUR.
