# Supplementary figures and images for: RDUR, a lncRNA, Promotes Innate Antiviral Responses and Provides Feedback Control of NF-κB Activation
Source: Front Immunol. 2021 May 14;12:672165. doi: 10.3389/fimmu.2021.672165 (PMC8160526; doi:10.3389/fimmu.2021.672165)

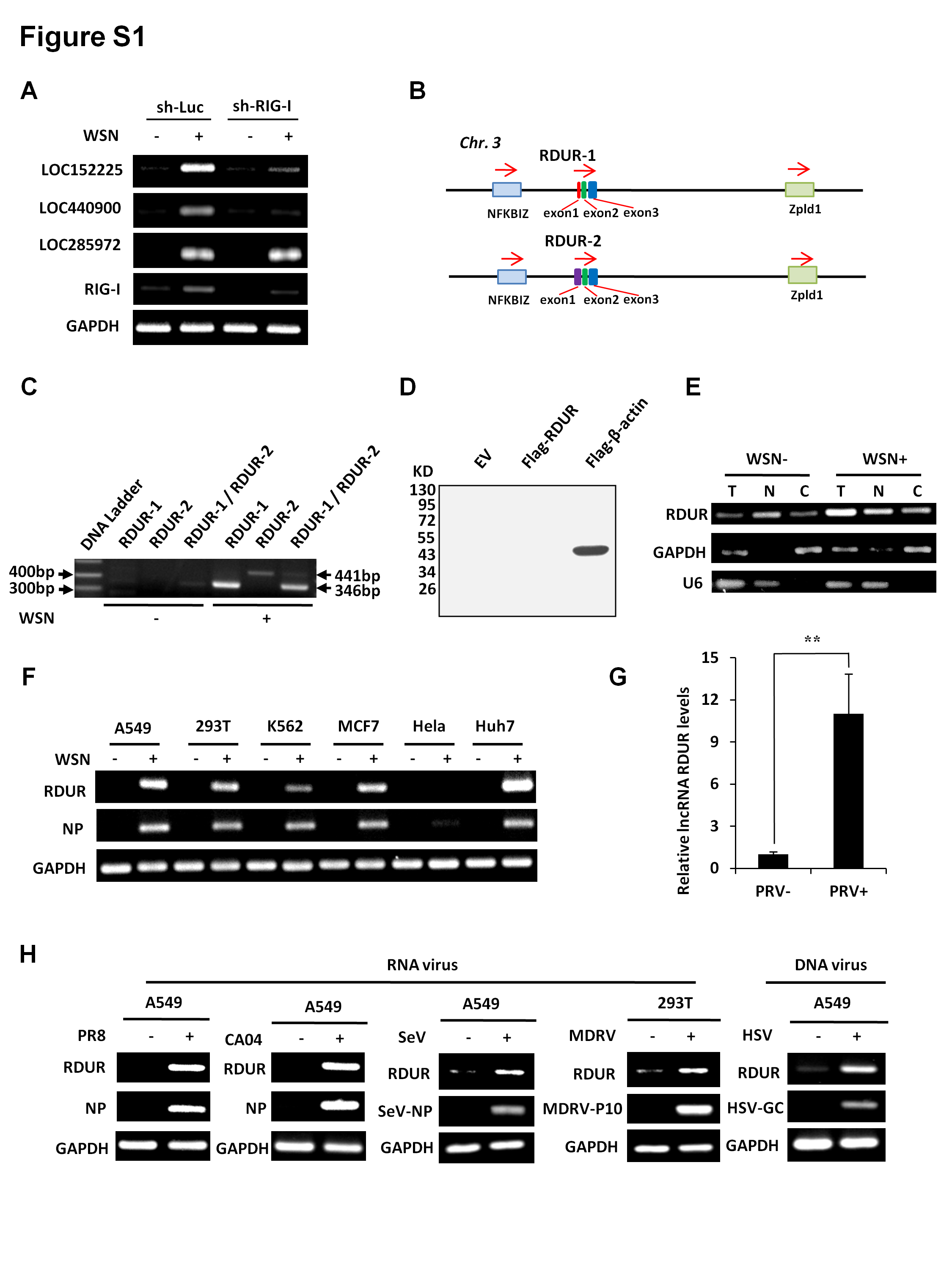

Supplement: Supplementary Figure 1 — Human RDUR is identified as a new lncRNA whose expression is greatly upregulated by influenza virus infection. (A) A549 cells expressing shRNAs targeting RIG-I or luciferase control were infected with or without WSN for 16 h, and the expression of the three lncRNAs was examined by RT-PCR. (B) Shown is a paradigm of the genomic location of lncRNA gene RDUR and the relationship with the genes NFKBIZ (light blue) and Zpld1 (light green). (C) Shown is the abundance of the two isoforms of RDUR examined by RT-PCR. (D) lncRNA RDUR or cDNA of actin (as control) was subcloned into the pFLAG-CMV-5a vector with Flag tag. A549 cell lines stably expressing actin, RDUR or empty vector (EV) were generated and analyzed by Western blotting using Flag antibody. (E) A549 cells were fractionated into nuclear and cytoplasmic fractions. RT-PCR was performed to examine the levels of RDUR, nuclear control transcript (U6) and cytoplasmic control transcript (glyceraldehyde 3-phosphate dehydrogenase, GAPDH) in the fractions. T: total RNA, N: nuclear RNA, C: cytoplasmic RNA. Shown are representative results from three independent experiments. (F) Human cell lines were infected with or without WSN (moi=1) for 16 h, and RDUR was examined by RT-PCR as described in Figure 1F . Shown are representative RT-PCR results from three independent experiments. (G) RDUR expression was detected by qRT-PCR in A549 cells infected with PRV virus. Shown are representative data from three independent experiments. (H) RDUR expression was examined by RT-PCR in indicated cells infected with different viruses as described in Figure 1G . Shown are representative RT-PCR results from three independent experiments. Related to Figure 1 . [file Image_1.tif]

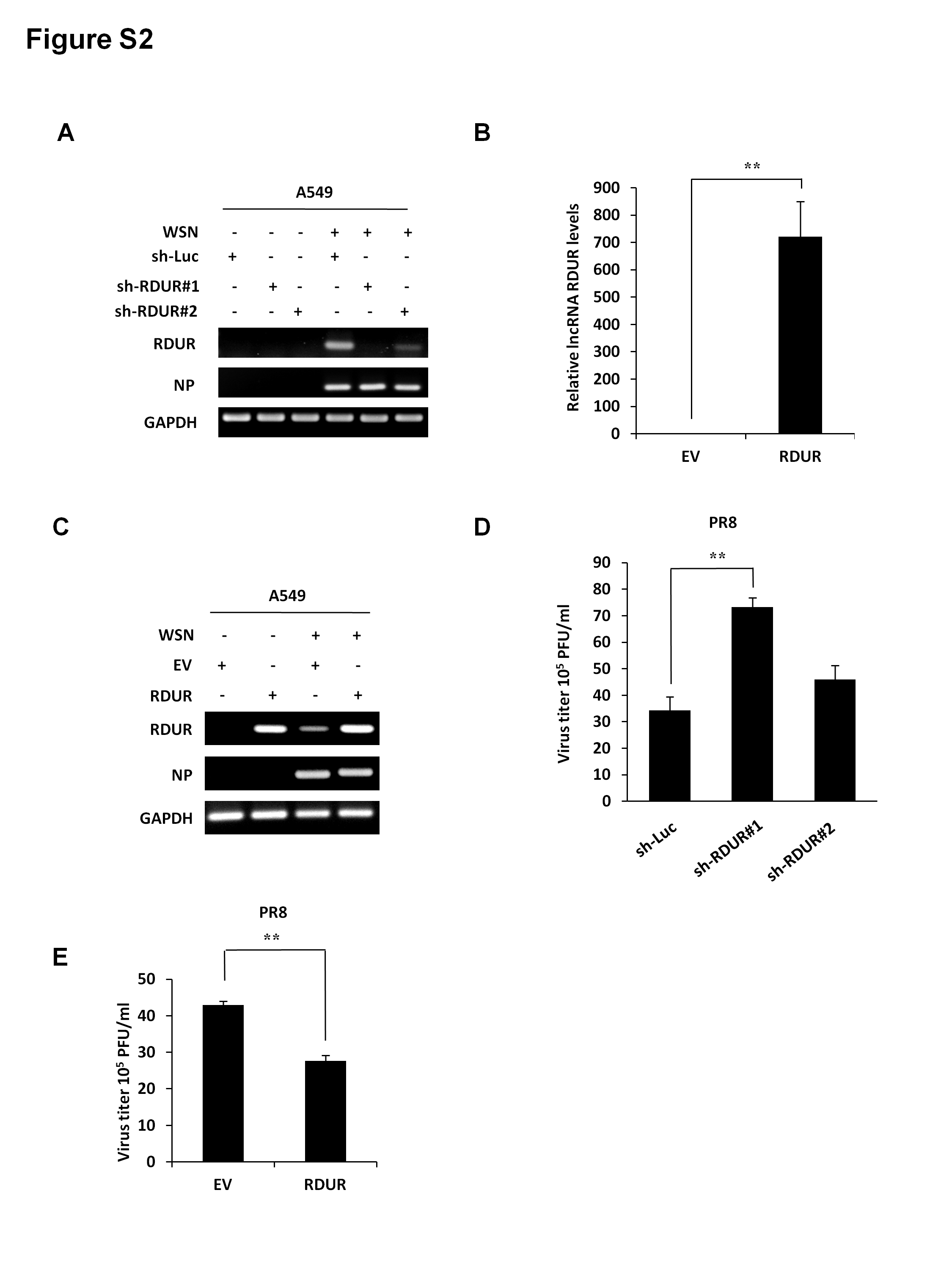

Supplement: Supplementary Figure 2 — Altering RDUR expression impacts IAV replication in A549 cells. (A) The efficiency of RDUR knockdown was determined by RT-PCR in control and WSN infected A549 cells. (B) The efficiency of RDUR overexpression was determined by qRT-PCR in A549 cells. Plotted are the average levels from three independent experiments. The error bars represent the SD, **P < 0.01. (C) The efficiency of RDUR overexpression was determined by RT-PCR in control and WSN infected A549 cells. (D) RDUR knockdown A549 cells were infected with PR8 for 16 h and the virus titers were measured by plaque assay. Plotted are the average levels from three independent experiments. Data are shown as means ± SD, **P < 0.01. (E) A549 cells overexpressing RDUR were infected by PR8 virus for 16 h and the titers in the supernatants of the culture medium were measured by plaque assay. Data are shown as means ± SD, **P < 0.01. Related to Figure 2 . [file Image_2.tif]

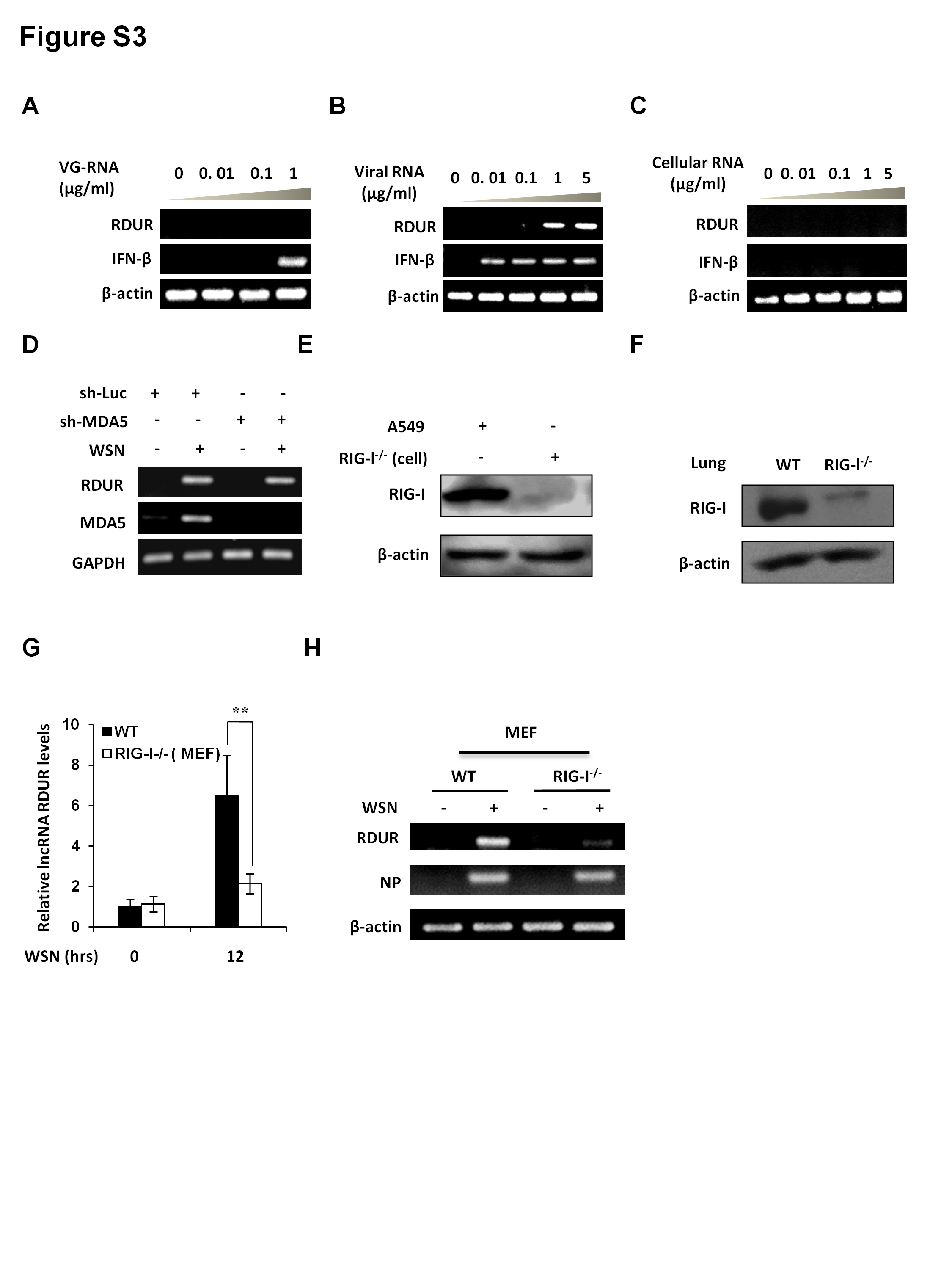

Supplement: Supplementary Figure 3 — IAV-induced robust expression of RDUR is RIG-I dependent both in vitro and in vivo. (A) A549 cells were transfected with indicated amount of WSN genomic RNA (VG-RNA) using Lipofectamine 2000. Effect of VG-RNA on the expression of RDUR and IFN-β was determined by RT-PCR. (B) Different amounts of total RNA (named as Viral RNA) isolated from A549 cells infected with the IAV were transfected into native A549 cells using Lipofectamine 2000. The expression levels of RDUR and IFN-β in the transfected A549 cells were examined by RT-PCR. (C) Different amounts of total RNA (named as Cellular RNA) from uninfected A549 cells were transfected into native A549 cells using Lipofectamine 2000 and the expression of RDUR and IFN-β in the transfected cells was examined by RT-PCR. (D) A549 cell lines stably expressing shRNAs targeting MDA5 was generated. Then, the cells were infected with WSN for 16 h, and the expression of RDUR was examined by RT-PCR. (E) RIG-I knockout A549 cell line was generated and examined by Western blotting with indicated antibodies. (F) WT and RIG-I knockout mice were sacrificed and the lungs were dissected and lysed, followed by Western blotting to detect the RIG-I level. (G, H) MEF cells derived from WT and RIG-I knockout mice were infected with WSN for 16 h, and the expression of RDUR was detected by qRT-PCR (K) and RT-PCR(L), respectively. Shown are representative data from three independent experiments. The error bars represent the SD. **P < 0.01. Related to Figure 3 . [file Image_3.tif]

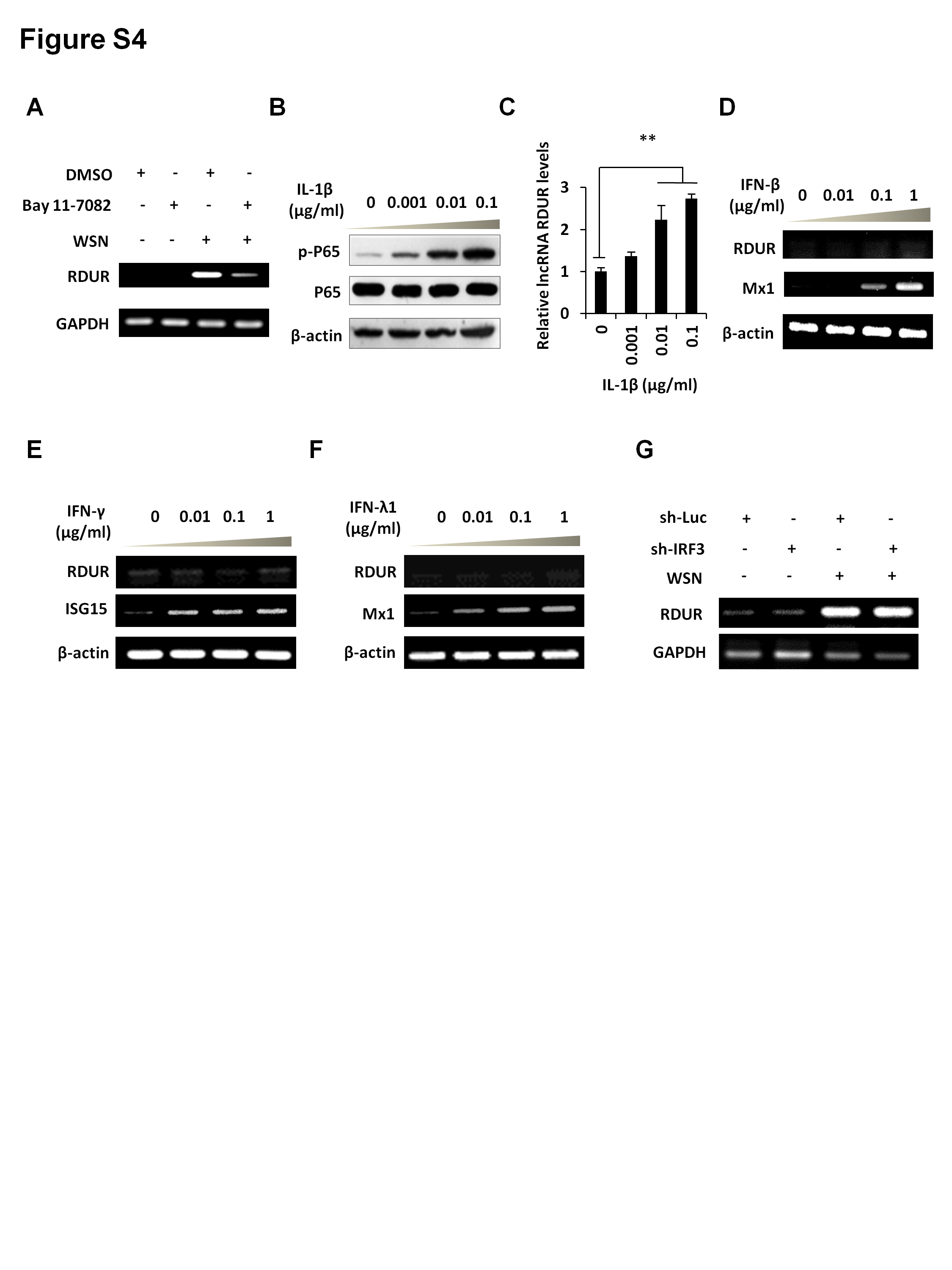

Supplement: Supplementary Figure 4 — IAV-induced RDUR expression is regulated by NF-κB but not IRF3 and IFNs. (A) A549 cells were treated with Bay 11-7082 as described in Figure 4C and the RNA levels of RDUR were determined by RT-PCR. (B, C) A549 cells were stimulated with IL-1β at indicated concentrations for 180 min. The activation of NF-κB was confirmed by Western blotting (B) and the RNA levels of RDUR were examined by qRT-PCR (C). (D–F) A549 cells were treated with different IFNs as described in Figures 4H–J . The expression levels of RDUR and ISGs (Mx1 or ISG15) were examined by RT-PCR. (G) IRF3 knockdown or control cells were infected with or without WSN for 16 h, followed by RT-PCR to detect RDUR levels. Related to Figure 4 . [file Image_4.tif]

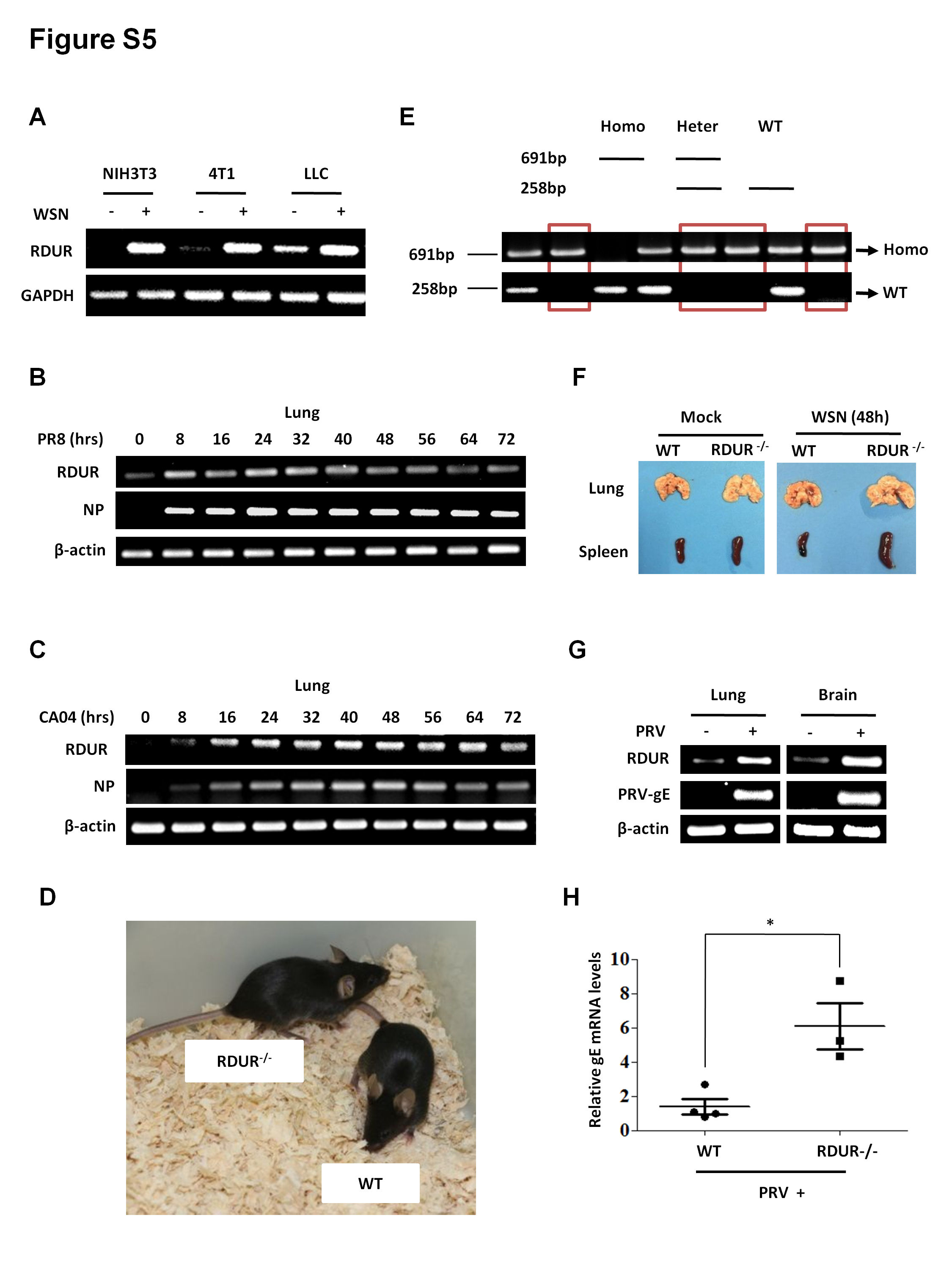

Supplement: Supplementary Figure 5 — In vivo studies of mRDUR in mice. (A) The expression levels of mRDUR in three mouse cell lines infected with WSN for 16 h were examined by RT-PCR. (B, C) The expression levels of mRDUR in C57BL/6J mice infected with influenza virus PR8 (B) or CA04 (C) at indicated time points were examined by RT-PCR. (D) Shown is a representative photograph of RDUR knockout C57BL/6J and WT mice used in this study. (E) The RDUR knockout mice were generated and genotyped by PCR. Shown is genotyping of RDUR knockout mice: WT, Hetero and Homo represent wild type, heterozygous and homozygous littermates, respectively. Homozygous RDUR knockout mice were indicated by red rectangles. (F) mRDUR knockout and control mice were intranasally inoculated with WSN or PBS for 48 h. Then mice were sacrificed and the lungs and spleens were collected. Shown are representative images from three independent experiments. (G) WT mice were intramuscularly infected with Pseudorabies virus (PRV) for 2 days, and the mRDUR levels in the lungs and brains of mice were determined by RT-PCR. (H) WT and RDUR-/- mice were intramuscularly infected with PRV for 2 days, and the PRV gE mRNA levels were detected in the lungs of mice. Related to Figure 5 . [file Image_5.tif]

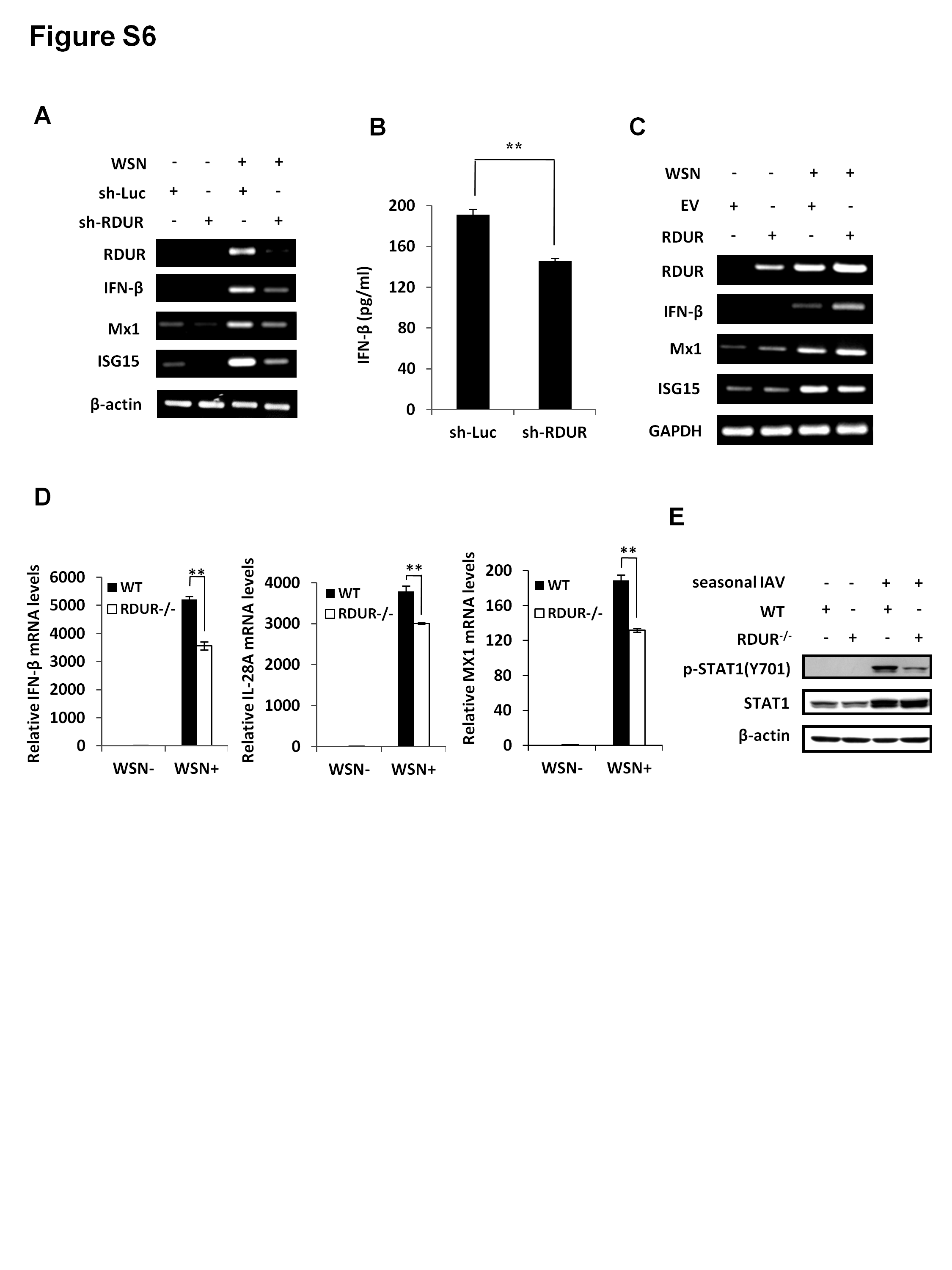

Supplement: Supplementary Figure 6 — RDUR regulates the expression of several critical antiviral genes. (A) The levels of RDUR, IFN-β, ISG15 and Mx1 in RDUR knockdown A549 cells infected with WSN were determined by RT-PCR. (B) Protein levels of IFN-β were determined by ELISA in RDUR knockdown A549 cells. Data are shown as means ± SD. **P < 0.01. (C) The levels of RDUR, IFN-β, ISG15 and Mx1 in RDUR overexpression A549 cells infected with WSN were determined by RT-PCR. (D) The mRNA levels of IFN-β, IL-28A and Mx1 in the lungs of WSN-infected mRDUR knockout or WT mice were determined by qRT-PCR. The error bars represent the SD, **P < 0.01. (E) mRDUR knockout and control mice were infected with a seasonal H1N1 influenza virus, and Western blotting was performed to detect related proteins. Related to Figure 6 . [file Image_6.tif]

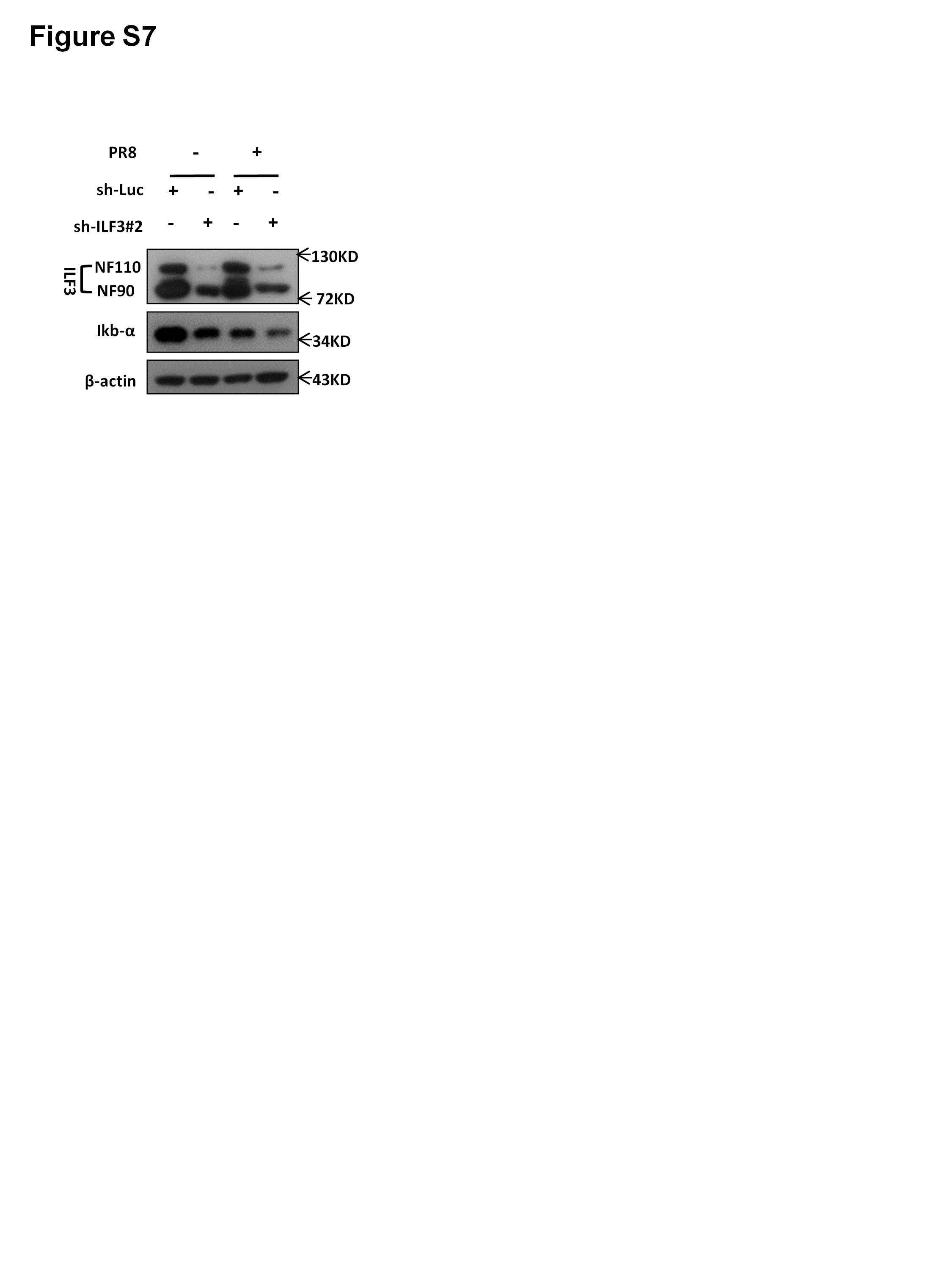

Supplement: Supplementary Figure 7 — RDUR interacts with ILF2 and ILF3 RNA binding proteins. A549 cell lines stably expressing specific shRNAs targeting ILF3 and luciferase (control) were infected with or without IAV and harvested at 14 hpi., followed by Western blotting with the indicated antibodies. [file Image_7.tif]

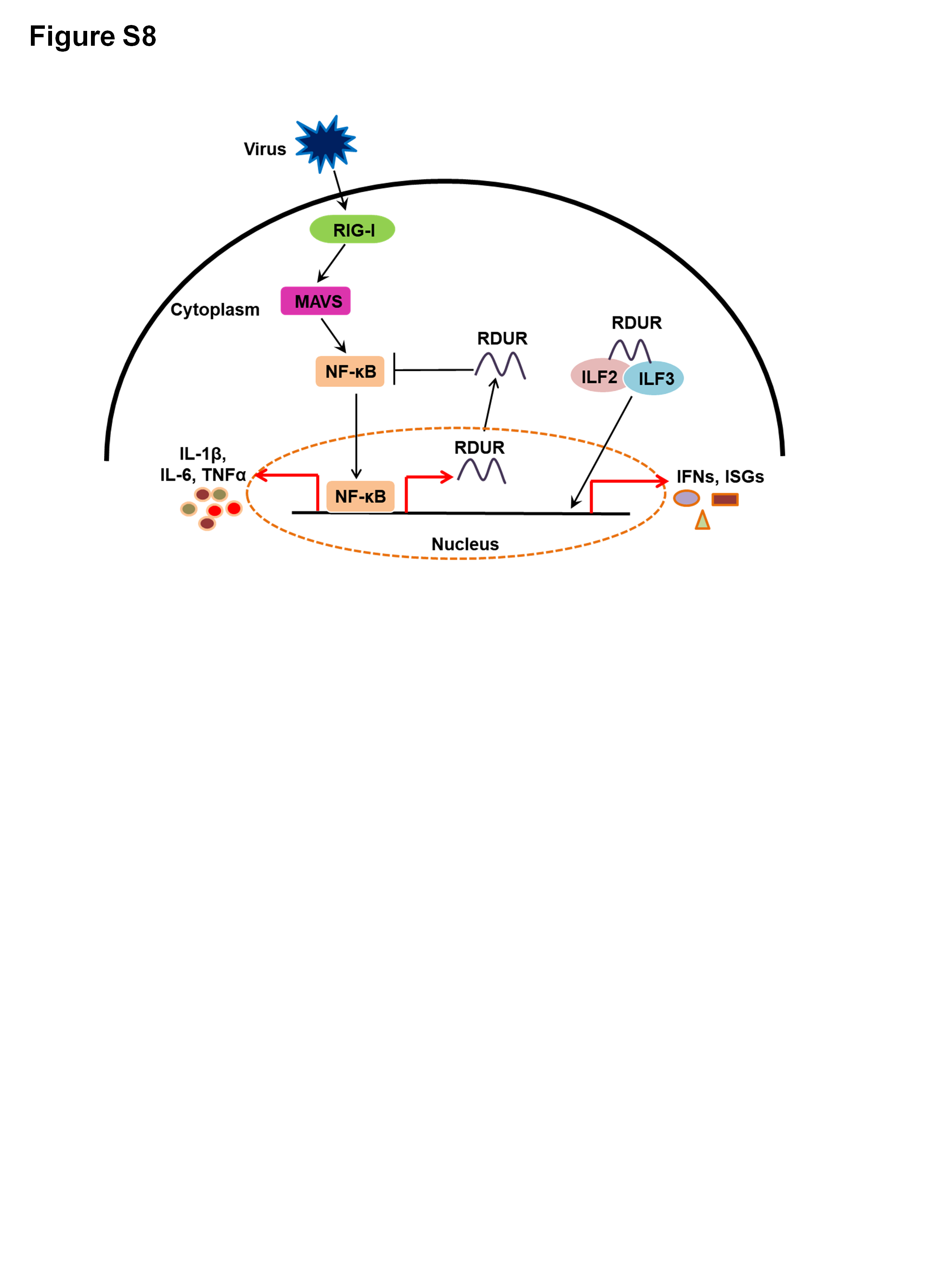

Supplement: Supplementary Figure 8 — The mechanisms of RDUR in antiviral innate immunity. The schematic diagram of RDUR. [file Image_8.tif]
