# Supplementary material for: RDUR, a lncRNA, Promotes Innate Antiviral Responses and Provides Feedback Control of NF-κB Activation
Source: Front Immunol. 2021 May 14;12:672165. doi: 10.3389/fimmu.2021.672165 (PMC8160526; doi:10.3389/fimmu.2021.672165)
Supplement: Supplementary file 9 [file Table_1.doc]

**Supplementary Table 1**

**Supplementary Table 1** Identification of proteins interacting with RDUR. RNA pull-down assay using biotinylated RDUR or control was performed and the precipitate was analyzed by mass spectrometry. Two proteins were identified as ILF2 and ILF3 that are highlighted in bold.

| Accession | Description | Score | Coverage |
| --- | --- | --- | --- |
| P60709 | Actin, cytoplasmic 1 OS=Homo sapiens GN=ACTB PE=1 SV=1 - [ACTB_HUMAN] | 50.50 | 63.73 |
| P00558 | Phosphoglycerate kinase 1 OS=Homo sapiens GN=PGK1 PE=1 SV=3 - [PGK1_HUMAN] | 25.58 | 42.93 |
| P62736 | Actin, aortic smooth muscle OS=Homo sapiens GN=ACTA2 PE=1 SV=1 - [ACTA_HUMAN] | 23.84 | 33.69 |
| **B4DY09**  **(ILF2)** | **Interleukin enhancer-binding factor 2 OS=Homo sapiens GN=ILF2 PE=1 SV=1 - [B4DY09_HUMAN]** | **19.41** | **40.91** |
| P02768 | Serum albumin OS=Homo sapiens GN=ALB PE=1 SV=2 - [ALBU_HUMAN] | 17.44 | 27.09 |
| H0Y8G5 | Heterogeneous nuclear ribonucleoprotein D0 (Fragment) OS=Homo sapiens GN=HNRNPD PE=1 SV=7 - [H0Y8G5_HUMAN] | 10.79 | 19.92 |
| Q5JS74 | Peripheral plasma membrane protein CASK OS=Homo sapiens GN=CASK PE=1 SV=1 - [Q5JS74_HUMAN] | 6.56 | 3.39 |
| P02452 | Collagen alpha-1(I) chain OS=Homo sapiens GN=COL1A1 PE=1 SV=5 - [CO1A1_HUMAN] | 4.01 | 8.13 |
| Q13148 | TAR DNA-binding protein 43 OS=Homo sapiens GN=TARDBP PE=1 SV=1 - [TADBP_HUMAN] | 3.99 | 11.35 |
| Q08554-2 | Isoform 1B of Desmocollin-1 OS=Homo sapiens GN=DSC1 - [DSC1_HUMAN] | 2.37 | 1.90 |
| P15924-2 | Isoform DPII of Desmoplakin OS=Homo sapiens GN=DSP - [DESP_HUMAN] | 1.82 | 1.50 |
| F8WD59 | 40S ribosomal protein SA (Fragment) OS=Homo sapiens GN=RPSA PE=1 SV=2 - [F8WD59_HUMAN] | 1.77 | 11.21 |
| P81605 | Dermcidin OS=Homo sapiens GN=DCD PE=1 SV=2 - [DCD_HUMAN] | 1.76 | 10.00 |
| A6NNT2 | Uncharacterized protein C16orf96 OS=Homo sapiens GN=C16orf96 PE=4 SV=3 - [CP096_HUMAN] | 0.00 | 1.58 |
| Q01105-3 | Isoform 3 of Protein SET OS=Homo sapiens GN=SET - [SET_HUMAN] | 0.00 | 4.91 |
| B3KUB4 | Carbonic anhydrase 12 OS=Homo sapiens GN=CA12 PE=1 SV=1 - [B3KUB4_HUMAN] | 0.00 | 3.18 |
| B5MCX3 | Septin-2 OS=Homo sapiens GN=SEPT2 PE=1 SV=1 - [B5MCX3_HUMAN] | 0.00 | 11.53 |
| C9JN71 | Zinc finger protein 878 OS=Homo sapiens GN=ZNF878 PE=3 SV=2 - [ZN878_HUMAN] | 0.00 | 1.69 |
| C9JPI2 | Junction plakoglobin (Fragment) OS=Homo sapiens GN=JUP PE=1 SV=1 - [C9JPI2_HUMAN] | 0.00 | 34.88 |
| C9JYW2 | Nucleolin (Fragment) OS=Homo sapiens GN=NCL PE=1 SV=1 - [C9JYW2_HUMAN] | 0.00 | 6.38 |
| E7EUT5 | Glyceraldehyde-3-phosphate dehydrogenase OS=Homo sapiens GN=GAPDH PE=1 SV=1 - [E7EUT5_HUMAN] | 0.00 | 14.62 |
| P23526-2 | Isoform 2 of Adenosylhomocysteinase OS=Homo sapiens GN=AHCY - [SAHH_HUMAN] | 0.00 | 10.40 |
| G5E9A7 | Dystrophia myotonica WD repeat-containing protein OS=Homo sapiens GN=DMWD PE=1 SV=1 - [G5E9A7_HUMAN] | 0.00 | 9.40 |
| H3BR62 | Cancer/testis antigen 62 OS=Homo sapiens GN=CT62 PE=4 SV=1 - [H3BR62_HUMAN] | 0.00 | 42.86 |
| O43157-3 | Isoform 3 of Plexin-B1 OS=Homo sapiens GN=PLXNB1 - [PLXB1_HUMAN] | 0.00 | 4.25 |
| O75874 | Isocitrate dehydrogenase [NADP] cytoplasmic OS=Homo sapiens GN=IDH1 PE=1 SV=2 - [IDHC_HUMAN] | 0.00 | 14.73 |
| P02656 | Apolipoprotein C-III OS=Homo sapiens GN=APOC3 PE=1 SV=1 - [APOC3_HUMAN] | 0.00 | 16.16 |
| P47929 | Galectin-7 OS=Homo sapiens GN=LGALS7 PE=1 SV=2 - [LEG7_HUMAN] | 0.00 | 16.18 |
| Q00577 | Transcriptional activator protein Pur-alpha OS=Homo sapiens GN=PURA PE=1 SV=2 - [PURA_HUMAN] | 0.00 | 3.11 |
| Q01085-2 | Isoform 2 of Nucleolysin TIAR OS=Homo sapiens GN=TIAL1 - [TIAR_HUMAN] | 0.00 | 2.30 |
| Q02413 | Desmoglein-1 OS=Homo sapiens GN=DSG1 PE=1 SV=2 - [DSG1_HUMAN] | 0.00 | 4.96 |
| Q14117 | Dihydropyrimidinase OS=Homo sapiens GN=DPYS PE=1 SV=1 - [DPYS_HUMAN] | 0.00 | 5.78 |
| O75369-2 | Isoform 2 of Filamin-B OS=Homo sapiens GN=FLNB - [FLNB_HUMAN] | 194.93 | 48.53 |
| P21333-2 | Isoform 2 of Filamin-A OS=Homo sapiens GN=FLNA - [FLNA_HUMAN] | 157.80 | 55.02 |
| Q08211 | ATP-dependent RNA helicase A OS=Homo sapiens GN=DHX9 PE=1 SV=4 - [DHX9_HUMAN] | 129.25 | 49.13 |
| P11142 | Heat shock cognate 71 kDa protein OS=Homo sapiens GN=HSPA8 PE=1 SV=1 - [HSP7C_HUMAN] | 104.29 | 52.48 |
| **Q12906-5**  **(ILF3)** | **Isoform 5 of Interleukin enhancer-binding factor 3 OS=Homo sapiens GN=ILF3 - [ILF3_HUMAN]** | **62.18** | **47.97** |
| P52272-2 | Isoform 2 of Heterogeneous nuclear ribonucleoprotein M OS=Homo sapiens GN=HNRNPM - [HNRPM_HUMAN] | 61.08 | 47.90 |
| P07900 | Heat shock protein HSP 90-alpha OS=Homo sapiens GN=HSP90AA1 PE=1 SV=5 - [HS90A_HUMAN] | 54.18 | 31.97 |
| P08238 | Heat shock protein HSP 90-beta OS=Homo sapiens GN=HSP90AB1 PE=1 SV=4 - [HS90B_HUMAN] | 50.80 | 37.98 |
| P12956 | X-ray repair cross-complementing protein 6 OS=Homo sapiens GN=XRCC6 PE=1 SV=2 - [XRCC6_HUMAN] | 40.09 | 38.26 |
| Q00839 | Heterogeneous nuclear ribonucleoprotein U OS=Homo sapiens GN=HNRNPU PE=1 SV=6 - [HNRPU_HUMAN] | 38.87 | 30.79 |
| P54652 | Heat shock-related 70 kDa protein 2 OS=Homo sapiens GN=HSPA2 PE=1 SV=1 - [HSP72_HUMAN] | 29.57 | 17.53 |
| P67809 | Nuclease-sensitive element-binding protein 1 OS=Homo sapiens GN=YBX1 PE=1 SV=3 - [YBOX1_HUMAN] | 28.46 | 43.52 |
| P0DMV9 | Heat shock 70 kDa protein 1B OS=Homo sapiens GN=HSPA1B PE=1 SV=1 - [HS71B_HUMAN] | 26.11 | 21.06 |
| P38646 | Stress-70 protein, mitochondrial OS=Homo sapiens GN=HSPA9 PE=1 SV=2 - [GRP75_HUMAN] | 26.10 | 30.19 |
| P19338 | Nucleolin OS=Homo sapiens GN=NCL PE=1 SV=3 - [NUCL_HUMAN] | 26.08 | 24.93 |
| P11940 | Polyadenylate-binding protein 1 OS=Homo sapiens GN=PABPC1 PE=1 SV=2 - [PABP1_HUMAN] | 24.15 | 25.63 |
| Q96AE4 | Far upstream element-binding protein 1 OS=Homo sapiens GN=FUBP1 PE=1 SV=3 - [FUBP1_HUMAN] | 23.34 | 31.99 |
| O60506-2 | Isoform 2 of Heterogeneous nuclear ribonucleoprotein Q OS=Homo sapiens GN=SYNCRIP - [HNRPQ_HUMAN] | 22.06 | 22.96 |
| P42704 | Leucine-rich PPR motif-containing protein, mitochondrial OS=Homo sapiens GN=LRPPRC PE=1 SV=3 - [LPPRC_HUMAN] | 21.99 | 12.12 |
| P16989-2 | Isoform 2 of Y-box-binding protein 3 OS=Homo sapiens GN=YBX3 - [YBOX3_HUMAN] | 20.85 | 28.05 |
| Q9NZB2 | Constitutive coactivator of PPAR-gamma-like protein 1 OS=Homo sapiens GN=FAM120A PE=1 SV=2 - [F120A_HUMAN] | 17.78 | 13.60 |
| P11021 | 78 kDa glucose-regulated protein OS=Homo sapiens GN=HSPA5 PE=1 SV=2 - [GRP78_HUMAN] | 13.13 | 6.42 |
| Q15393 | Splicing factor 3B subunit 3 OS=Homo sapiens GN=SF3B3 PE=1 SV=4 - [SF3B3_HUMAN] | 11.56 | 5.01 |
| Q15020 | Squamous cell carcinoma antigen recognized by T-cells 3 OS=Homo sapiens GN=SART3 PE=1 SV=1 - [SART3_HUMAN] | 10.16 | 17.55 |
| Q14247-3 | Isoform 3 of Src substrate cortactin OS=Homo sapiens GN=CTTN - [SRC8_HUMAN] | 9.80 | 14.42 |
| P81605 | Dermcidin OS=Homo sapiens GN=DCD PE=1 SV=2 - [DCD_HUMAN] | 8.16 | 42.73 |
| Q9BUJ2-4 | Isoform 4 of Heterogeneous nuclear ribonucleoprotein U-like protein 1 OS=Homo sapiens GN=HNRNPUL1 - [HNRL1_HUMAN] | 7.96 | 24.07 |
| P55265-5 | Isoform 5 of Double-stranded RNA-specific adenosine deaminase OS=Homo sapiens GN=ADAR - [DSRAD_HUMAN] | 7.67 | 8.92 |
| A0A0B4J1R6 | Transketolase OS=Homo sapiens GN=TKT PE=1 SV=1 - [A0A0B4J1R6_HUMAN] | 7.57 | 16.85 |
